# Inactivation of antibiotic-resistant bacteria and antibiotic-resistance genes in wastewater streams: Current challenges and future perspectives

**DOI:** 10.3389/fmicb.2022.1100102

**Published:** 2023-01-16

**Authors:** Thabang B. M. Mosaka, John O. Unuofin, Michael O. Daramola, Chedly Tizaoui, Samuel A. Iwarere

**Affiliations:** ^1^Department of Chemical Engineering, Faculty of Engineering, Built Environment and Information Technology, University of Pretoria, Pretoria, South Africa; ^2^Water and Resources Recovery Research Lab, Department of Chemical Engineering, Faculty of Science and Engineering, Swansea University, Swansea, United Kingdom

**Keywords:** antibiotic-resistant bacteria, antibiotic-resistance genes, wastewater, disinfection method, cold atmospheric plasma

## Abstract

The discovery of antibiotics, which was once regarded as a timely medical intervention now leaves a bitter aftertaste: antimicrobial resistance (AMR), due to the unregulated use of these compounds and the poor management receiving wastewaters before discharge into pristine environments or the recycling of such treated waters. Wastewater treatment plants (WWTPs) have been regarded a central sink for the mostly unmetabolized or partially metabolised antibiotics and is also pivotal to the incidence of antibiotic resistance bacteria (ARBs) and their resistance genes (ARGs), which consistently contribute to the global disease burden and deteriorating prophylaxis. In this regard, we highlighted WWTP-antibiotics consumption-ARBs-ARGs nexus, which might be critical to understanding the epidemiology of AMR and also guide the precise prevention and remediation of such occurrences. We also discovered the unsophistication of conventional WWTPs and treatment techniques for adequate treatment of antibiotics, ARBs and ARGs, due to their lack of compliance with environmental sustainability, then ultimately assessed the prospects of cold atmospheric plasma (CAP). Herein, we observed that CAP technologies not only has the capability to disinfect wastewater polluted with copious amounts of chemicals and biologicals, but also have a potential to augment bioelectricity generation, when integrated into bio electrochemical modules, which future WWTPs should be retrofitted to accommodate. Therefore, further research should be conducted to unveil more of the unknowns, which only a snippet has been highlighted in this study.

## 1. Introduction

Antibiotics are used for the inhibition or complete destruction of bacteria that cause infections in humans and animals (Yuan et al., 2015; Duijkeren et al., 2018; Li and Gu, 2019; Sarangapani et al., 2019) and are also widely used in cancer treatment and in some regions, as growth promotion agents (Sarangapani et al., 2019). There has been a global increase in the consumption of antibiotics because these drugs are becoming more affordable and accessible (Genthe et al., 2020). Since most antibiotics are not completely metabolised by humans and animals, they are often ejected as common components of wastewater (Fadare and Okoh, 2021a) where they induce increased antibiotic resistance of common bacteria, and the gradual development of broad-spectrum antibiotic-resistant genes (Huang et al., 2013; Yuan et al., 2015). This further explains the high concentration of ARBs and ARGs that are often reticulated into wastewater treatment plants (WWTPs) from the sewage systems of households, healthcare services, antibiotic manufacturing facilities, agricultural activities and animal feedlots (Ekwanzala et al., 2018; Ben et al., 2019; Rodriguez-Molina et al., 2019). WWTPs have been principally designed to remove nutrients and reduce bacterial load to certain acceptable limits; regrettably, their optimum performances do not remove ARBs and ARGs (Fadare and Okoh, 2021b). When the conventional disinfection processes such as chlorination, UV irradiation, and ozone oxidation are applied, a great fraction of ARBs dies, while others enter a state of dormancy due to stress, and are resuscitated when the stressors are released. Disinfectants such as chlorine tend to have a selective effect on ARGs, decreasing abundance of genes (gene copies per mL of sample) while the prevalence of the gene (gene copies per total bacteria) increases (Manaia et al., 2018; Chen et al., 2020b). Sometimes disinfection processes may kill the bacteria by destroying its DNA or the cellular structure, but ARGs may still persist for a long time in the cell debris and in the environment. Both intracellular (i-) and extracellular (e-) ARGs eventually transfer and adapt into new bacteria, leading to the inception and genetic transformation across bacteria and the development of antibiotic resistance (Yuan et al., 2015; Sarangapani et al., 2019; Chen et al., 2020b; Jin et al., 2020). These ARBs and ARGs present in WWTPs are released into outgoing environmental systems such as rivers and reservoirs (Rodriguez-Molina et al., 2019). Wastewater has been previously discussed as both a resource and a problem (Unuofin, 2020); the compelled reuse of treated wastewater due to overstretched natural water resources in water-stressed countries further increases the risks of ARBs and ARGs exposure.

WWTPs have been observed to be direct key reservoir of ARBs and ARGs associated with human infection as high concentrations of ARBs and ARGs have been detected in therein, worldwide Moreover, investigations have shown that patients with infections caused by bacteria regarded as critical by the World Health Organisation (WHO) consume more health-care resources, because they are more at risk of worse clinical outcomes and death than patients infected with non-resistant strains of the same bacteria (World Health Organisation, 2020). Therefore, there is a need to monitor and control these ARBs and ARGs in the WWTPs, which might be instrumental in preventing their contact with pristine water bodies as well humans and animals (Huang et al., 2013; Ekwanzala et al., 2018; Wang et al., 2020). To our knowledge, no well-documented strategy is in place to prevent the movement of the ARBs and ARGs in the environment. An alternative tool for both water treatment and wastewater reclamation and reuse is Advanced Oxygenation Processes (AOPs) which breaks down organic matter while inactivating ARBs and ARGs (Umar, 2022). Considering that conventional disinfectants like chlorine tend to be selective in what it actually oxidizes, AOPs produce reactive oxygen species (ROS) like the indiscriminate hydroxyl radical •OH (Foster, 2017; Chen Y. et al., 2020). The primary regimes of AOPs-driven disinfection include the destruction of cell wall, cell membrane, enzymes, and intracellular genetic material (Chen Y. et al., 2020). The interest in the role of •OH is that it has an oxidation potential (2.08 V) that is higher than the conventional disinfectants, chlorine (1.36 V) and ozone (2.07 V), and it can damage DNA (Foster, 2017; Sharma et al., 2019; Rekhate and Srivastava, 2020; Azuma et al., 2022). The •OH radical has diverse impact on normal protein structure which is one of the primary targets in bacteria during disinfection, including oxidation of amino acids, modification of sulphur groups, etc., causing irreversible damages to cells and inactivation of ARBs and ARGs (Chen Y. et al., 2020).

The inadequate wastewater treatment (that can be assisted by AOPs) coupled with poor data collation contribute to greater challenge of tackling antibiotic resistance (Genthe et al., 2020). This review thus elucidates the WWTP-antibiotics consumption-ARBs-ARGs nexus, which is an invaluable blueprint for managing ARBs and ARGs occurrences and also assessing the efficiency of WWTPs. The review also provides a commentary and analysis on the extant and the emerging treatment technologies, particularly cold atmospheric plasma (CAP), which is renowned for its environmental friendliness and swift response during operation.

### 1.1. Statement of significance

This paper provides a critical review on the efficiency of commonly used disinfection methods (e.g., chlorination, ozone and Ultraviolet (UV) irradiance) for the inactivation of antibiotic resistant bacteria (ARB) and antibiotic resistant genes (ARGs) wastewater treatment plants (WWTPs). It was observed that these disinfection methods alone were not able to completely inactivate the ARBs and ARGs. Enhanced removal efficiencies were only noticed when they were used alongside ultrafiltration, anaerobic membrane bioreactors, electrocoagulation, tertiary filtration and peracetic acid. Cold atmospheric plasma is suggested as an alternate all in one disinfection method, that generates intense UV radiation, shockwaves, reactive oxygen species and reactive nitrogen species that prevent procreation of cells and the spread of ARBs and ARGs in the environment.

### 1.2. Literature synthesis

The PRISMA guidelines were used for this systematic review and compilation of removal efficiencies of ARBs and ARGs by actual wastewater treatment plants WWTPs (Moher et al., 2010). The literature search was performed using seven online databases: PubMed Database, EBSCOhost Online Research Databases, MEDLINE, ISI Web of Knowledge, African Journals Online, and Scopus in August 2022. Predefined terms such as (Antibiotic OR Resistance OR Bacteria OR ARBs OR Gene OR ARG OR WWTP OR Influent OR Effluent OR Inflow OR Outflow) AND (Treatment OR Disinfection Methods) were used to retrieve relevant articles published from March 1, 2021 to August 31, 2022. Supplementary Figure S1 summarizes the steps taken to conduct the literature search and selection. The first step entailed removing duplicate articles that were found in the seven databases. The remaining articles were screened based on their title and abstract. Full-text articles were read and screened. The remaining full-text articles were read and included in the review. Only articles that contain information on the detection of ARBs and ARGs in the influent and effluent of WWTPs were included in this review, regardless of the types of biological processes that were applied for quantification. Articles referring to ARGs detected from viruses and other micro-organisms other than bacteria were also excluded.

## 2. ARBs and ARGs in WWTPs

### 2.1. Incidence of ARBs and ARGs in wastewater

While there might be disagreeing accounts regarding the particular origin of AMR because the biochemical and molecular basis of such phenomenon was yet to be established in early studies (Hawkey, 1998; Moher et al., 2010), advancement in research has evinced not only its incidence but also suggested the rapidity in its evolution. The discovery of antibiotics has been observed as one of the most critically important healthcare interventions of the 20th century, where they were observed to reduce disease burden using different mechanisms against bacteria, such as inhibiting synthesis of the cell wall, depolarizing the cell membrane, inhibiting synthesis of the protein, inhibiting synthesis of the nucleic acid and inhibiting metabolic pathways in bacteria (Reygaert, 2018). Antibiotics have thereafter been abused, misused, overused and continually released into natural bodies through WWTPs, which are considered as sinks of major antibiotic reticulation pathways, such as households, aquaculture, healthcare facilities, antibiotic manufacturing facilities, agricultural activities, animal feedlots and slaughterhouses (Ekwanzala et al., 2018; Ben et al., 2019; Rodriguez-Molina et al., 2019). Correspondingly, numerous reviews have been able to identify the most predominantly consumed or utilised antibiotic categories as: macrolides, sulfonamides, trimethoprim, quinolones, tetracyclines, due to their prevalence in WWTPs and groundwater (Nnadozie et al., 2017; Wang et al., 2020; Noor et al., 2021) further reports a list of 16 antibiotic families based on their corresponding ARGs extrapolated from analysis of five continents. WWTPs have to capacity to hold, daily, phenomenal volumes of wastewater containing cocktails of chemical contaminants and organic matter, which are consistently biotransformed by denizen microorganisms (both beneficial and harmful). However, despite considerable achievement by these plants in reducing major pollutants through a combination of physicochemical and biological techniques, several reports have highlighted the surreptitiousness and subsequent evasion of certain classes of microcontaminants, especially antibiotics, as well as some ARBs and ARGs during such treatment processes (Unuofin, 2020; Wang et al., 2020; Zainab et al., 2020; Noor et al., 2021). Once in WWTPs, the persistent interaction of microbial denizens with antibiotics under genial conditions, such as adequate nutrients levels, biofilms abundance and other physicochemical conditions might facilitate microbial tolerance and evolution in resistance to gradually increasing concentration of antibiotics. Innately, bacterial populations of WWTP matrices might derive tolerance and resistance through certain morpho-physiological, biochemical and molecular phenomena. Intrinsic resistance mechanisms that have been documented, so far, include reduced membrane permeability, induced modification of intracellular antibiotic target (i.e., protein, ribosome, etc.), structural modification of the antibiotic, thus inactivating it, secretion of exopolymeric substances (EPS) or biofilms to immobilize and annul the bioavailability of the foreign chemical, use of active drug efflux pumps which expel antibiotics from inside the bacteria before they reach the specific binding site and apply the antimicrobial activity and also the expression of constitutive and inducible genes, which have evolved over time, due to selection pressure and recombination (Reygaert, 2018; Magureanu et al., 2021).

**Figure 1:**
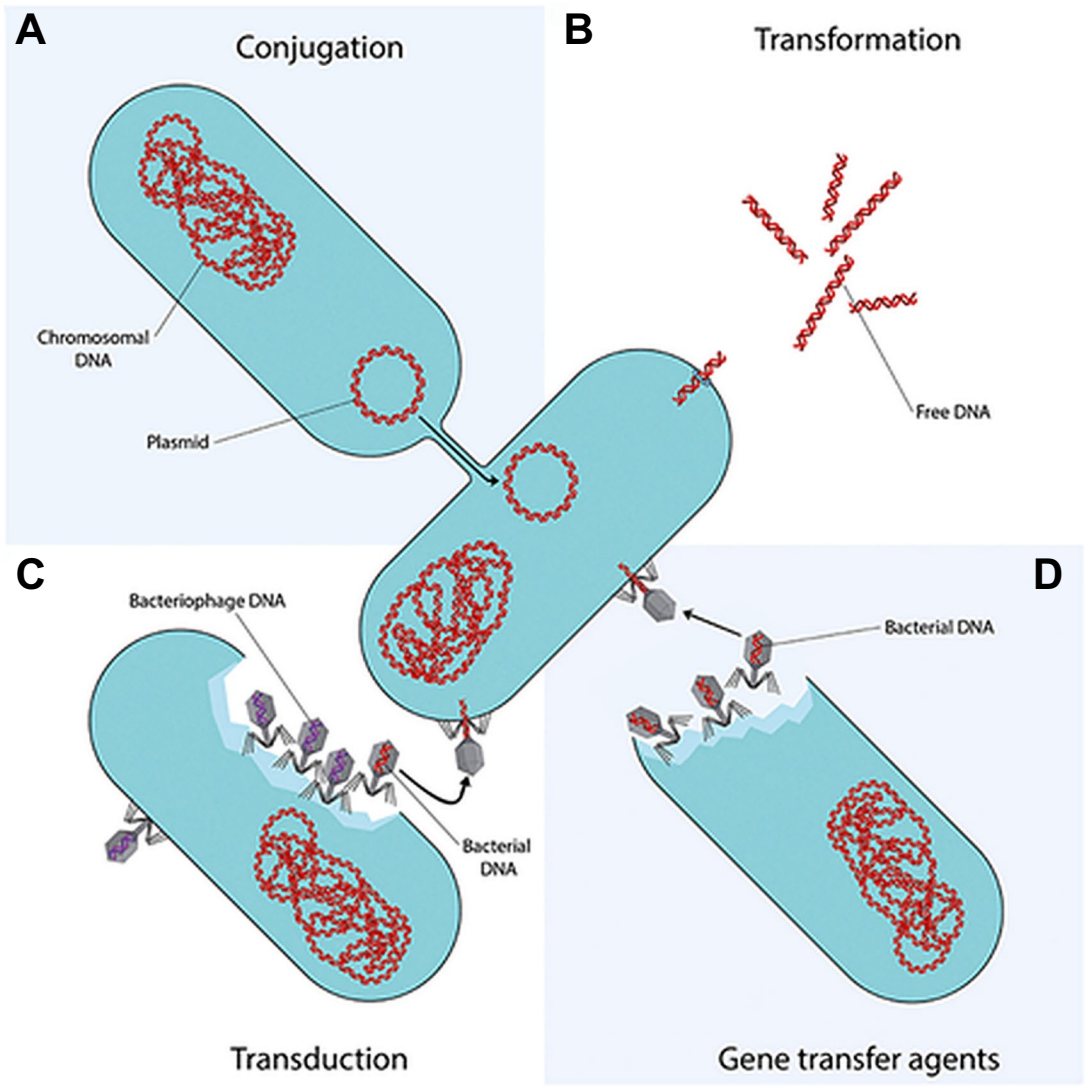
Horizontal gene transfer (HGT) of ARBs and ARGs (Von Wintersdorff et al., 2016). Conjugation is the direct transfer of DNA molecule known as a plasmid from a donor bacterium to a recipient bacterium, involving cell-to-cell contact between the two bacteria. Transduction is the transfer of DNA from a donor bacterium to a recipient bacterium, through viruses that infect bacteria, known as bacteriophages. Transformation is intra- and inter-species exchange of genetic information by uptake of naked DNA, released through cell lysis or actively excreted by some bacteria which can only be received by a competent bacterium. Following uptake and translocation to the cytoplasm, it is incorporated into the competent bacterium’s chromosome or into a plasmid (Koutsoumanis et al., 2021; Uluseker et al., 2021; Courti et al., 2022).

Amongst the aforementioned mechanisms, the genetic factor is considered the most critical due to its capability to constantly evolve to match up to the constantly improving antibiotic efficacies. Moreover, ARBs of WWTPs might be able to confer resistance status on the innocuous communities through horizontal gene transfer (Figure 1), and thereafter vertical transfer of recombinant DNA during proliferation. While HGT is considered to be a non-reproductive gene transfer whereby genetic material are dispersed between bacteria that do not have an offspring-parent relationship, HGT can transfer ARGs faster and effectively, which is why HGT is the most concerning transfer mechanism when it comes to AR spread in WWTPs (Uluseker et al., 2021; Courti et al., 2022). This has warranted a knee-jerk response regarding the constant surveillance of AMR, worldwide, assessment of extant as well emerging water and wastewater treatment techniques and technologies that would obliterate the threat posed by AMR.

### 2.2. Current global status of ARBs and ARGs occurrences in WWTPs

The immense pressure on and critical reduction and pollution of globally available freshwater withdrawals coupled with the increasing incidences of deteriorating prophylaxis, especially regarding bacterial infections, has necessitated a more focused look at the once overlooked pollution sinks: WWTPs. Ever since the earliest surveillance, there has been overwhelmingly consistent studies, worldwide, that report the occurrence of antibiotics, ARBs and ARGs in different WWTP matrices, thereby suggesting their pivotal role in the infection cycle. Therefore, understanding the current global trend on occurrence of the WWTP-antibiotics consumption-ARBs-ARGs nexus is germane to developing a framework for preventive and remediation measures. To this end, we wish to provide a robust account on the current trend by assessing reviews by Wang et al. (2020), Noor et al. (2021), Uluseker et al. (2021), Zhuang et al. (2021), Gao et al. (2022) and Wang et al. (2022) *inter alia*, where especial attention was conferred on WWTPs on different geographical regions worldwide. From the reviews, it was apparent that there was no phenomenal reduction in concentrations of antibiotics, ARBs and ARGs, between influent and effluents. This might be due to lipophilicity and hydrophobicity of antibiotics that reduce their liability to certain physicochemical and biological treatment. Although cells of resistant bacteria might be damaged during treatment, their free lying genetic materials remain a threat, which might be picked up by innocuous population in effluent and also downstream, thus rendering preceding treatment steps ineffective. The volatility and subsequent aerosolization of ARGs was observed as well, which could be a determinant in their evasion from treatment and transboundary movement to already treated effluent. It was also deduced that the type and abundance of ARGs in WWTP influents could be used to fingerprint the categories of antibiotics with unregulated use. To corroborate this, our observation of high ARGs concentrations in WWTPs of high-income and upper-middle-income regions as compiled by Wang et al. (2022), was in congruence with the outcomes of a global survey on consumption and usage of antibiotics, where Western Europe and East Asia consumed a daily doses (DDD) of 3,364 million and 4,413 million units, respectively. Some of the genes commonly detected in WWTPs in the studies examined include variants of *sul*, *tet*, *erm*, *mph*, *bla*, *qnr*, *msr*, *mex*, which confer resistance to sulfonamide, tetracycline, β-lactams, macrolides, quinolones, as well as multidrug resistance (Zhuang et al., 2021), thereby suggesting the accelerated use of this group of antibiotics, globally as discussed subsequently. Quinolones are excreted unchanged by urine and faeces into WWTPs (Pazda et al., 2019) but later eliminated *via* sorption to sludge as they are very hydrophilic compounds (Hendricks and Pool, 2012). The quinolones resistance genes, *qnr* (*qnrB, qnrD*, and *qnrS*) are however present in China (WWTP1 and WWTP2; Table 1) as they are propagated by HGT (Mao et al., 2015; Pazda et al., 2019).

**Table 1:** Concentration of genes in the influent and effluent of different WWTPs.

Genes	WWTP	Concentration (*copies / ml*) in the influent	Concentration (*copies / ml*) in the effluent	References
*ermB*	WWTP1	$9\;x\;10^{5}$	$2\;x\;10^{3}$	Mao et al., 2015
	WWTP2	$1\;x\;10^{6}$	$2\;x\;10^{5}$	Mao et al., 2015
	NGWRP	$1.2\;x\;10^{5}$	0	Pazda et al., 2019
	WRPF	$6.31\;x\;10^{5}$	$1\;x\;10^{2}$	Kappell et al., 2018
	WWTP5 (UV)	$5.37\;x\;10^{4}$ cell eqivalents/100ml	$3.75\;x\;10^{4}$ cell eqivalents/100ml	Jager et al., 2018
	WWTP5 (Ozone)	$5.37\;x\;10^{4}$ cell eqivalents/100ml	$1.01\;x\;10^{3}$ cell eqivalents/100ml	Jager et al., 2018
	WWTP5 (UV and Ozone)	$5.37\;x\;10^{4}$ cell eqivalents/100ml	$1.07\;x\;10^{3}$ cell eqivalents/100ml	Jager et al., 2018
*qnr*	WWTP1	$7\;x\;10^{4}$	$1\;x\;10^{3}$	Mao et al., 2015
	WWTP2	$2\;x\;10^{5}$	$9\;x\;10^{3}$	Mao et al., 2015
*sul*	WWTP1	$3\;x\;10^{7}$	$5\;x\;10^{5}$	Mao et al., 2015
	WWTP2	$9\;x\;10^{6}$	$6\;x\;10^{5}$	Mao et al., 2015
	WWTP3	$1.19\;x\;10^{8}$	$4.52\;x\;10^{6}$	Zhang et al., 2017
	WWTP4 (UV)	$1.86\;x\;10^{5}$	$2.5\;x\;10^{3}$	Chen et al., 2020a
	WWTP4 (UV and EC)	$1.86\;x\;10^{5}$	$2x\;10^{2}$	Chen et al., 2020a
	WWTP5 (UV)	$1.33\;x\;10^{6}$ cell eqivalents/100ml	$9.33\;x\;10^{5}$ cell eqivalents/100ml	Jager et al., 2018
	WWTP5 (Ozone)	$1.33\;x\;10^{6}$ cell eqivalents/100ml	$6.83\;x\;10^{4}$ cell eqivalents/100ml	Jager et al., 2018
	WWTP5(UV and Ozone)	$1.33\;x\;10^{6}$ cell eqivalents/100ml	$5.53\;x\;10^{4}$ cell eqivalents/100ml	Jager et al., 2018
	NGWRP	$1.55\;x\;10^{5}$	0	Wallmann et al., 2021
	WRP	$2\;x\;10^{6}$	$3\;x\;10^{1}$	Quach-Cu et al., 2018
	WRPF	$6.31\;x\;10^{6}$	$3.98\;x\;10^{3}$	Kappell et al., 2018
*tet*	WWTP1	$8\;x\;10^{5}$	$3\;x\;10^{4}$	Mao et al., 2015
	WWTP2	$2\;x\;10^{6}$	$3\;x\;10^{5}$	Mao et al., 2015
	WWTP3	$1.78\;x\;10^{8}$	$2.49\;x\;10^{7}$	Zhang et al., 2017
	WWTP4 (UV)	$3.18\;x\;10^{3}$	$1.2\;x\;10^{3}$	Chen et al., 2020a
	WWTP4 (UV and EC)	$3.18\;x\;10^{3}$	$1\;x\;10^{2}$	Chen et al., 2020a
	WRPF	$5.01\;x\;10^{5}$	$6.31\;x\;10^{1}$	Kappell et al., 2018
*bla_SHV/TEM_*	WRP	$8\;x\;10^{3}$	$6\;x\;10^{0}$	Quach-Cu et al., 2018
	WWTP5 (UV)	$1.22\;x\;10^{5}$ cell eqivalents/100ml	$1.83\;x\;10^{5}$ cell eqivalents/100ml	Jager et al., 2018
	WWTP5 (Ozone)	$1.22\;x\;10^{5}$ cell eqivalents/100ml	$1.1\;x\;10^{4}$ cell eqivalents/100ml	Jager et al., 2018
	WWTP5(UV and Ozone)	$1.22\;x\;10^{5}$ cell eqivalents/100ml	$1.12\;x\;10^{4}$ cell eqivalents/100ml	Jager et al., 2018

The RNA methyltransferase, *ermB*, which is located on the transposon (Pazda et al., 2019; Wallmann et al., 2021), is prevalent in Gram-positive enterococci and it confers resistance to critically important macrolide-lincosamine-streptogramin (MLS) antibiotics like erythromycin, azithromycin and clarithromycin (Wallmann et al., 2021). The ermB genes are present in China (WWTP1, WWWP2), Namibia (NGWRP), United States (WRPF) and Germany (WWTP5; Table 1).

Penicillin’s (ampicillin, amoxicillin and clavulanic acid) and cephalosporins (cefotaxime) are the main β**-**lactam antibiotics used for veterinary and human medicine (Defrancesco et al., 2017; Pazda et al., 2019). The instability of the β**-**lactam ring and its susceptibility to hydrolysis makes β**-**lactam antibiotics, especially penicillin, not easily detected in WWTPs (Pazda et al., 2019). Extended-spectrum β-lactamase (ESBL)-producing bacteria, including *E. coli*, are among the most commonly encountered multidrug-resistant (MDR) bacteria today and are frequently associated with a high mortality rate and prolonged hospitalisation. This may be because clinical *E. coli* isolates frequently co-carry multiple ESBL genes with varying hydrolysis spectra to different antibiotics, leading to treatment failure (Seyedjavadi et al., 2016; Gumede et al., 2021). The genes that confer resistances to ESBL is the *bla*_CTX-M_ (Bockelmann et al., 2009; Defrancesco et al., 2017), while the *bla*_TEM_ and *bla*_SHV_ genes confer resistance to the narrow-spectrum β-lactams (Defrancesco et al., 2017). Specific variants of these, such as bla_SHV-5_, have the ability to hydrolyze broad-spectrum cephalosporins and monobactams (Nzima et al., 2020). The *bla*_TEM_, *bla*_SHV_ and *bla*_CTX-M_ are located on the plasmid while the *ampC* (ampicillin resistant) can be found on the chromosome (Pazda et al., 2019). Total heterotrophic bacteria (THB) resistant to ampicillin was present in Italy (WWTP6, WWTP7, WWTP8) and *bla*_SHV/TEM_ was present in United States (WRP) and Germany (WWTP5; Table 1).

The most persistent antibiotics in the environment, with synergistic actions of the metabolite with other antibiotics which results in a longer degradation time of 60 days, are sulfamethoxazole and/or the sulfamethoxazole–trimethoprim (STX) combination (Hendricks and Pool, 2012; Genthe et al., 2020). Trimethoprim and sulphonamides (sulfamethoxazole, sulfadiazine, sulfachloropryridazine, sulfacetamide, sulfasalazine and acetylsulfamethoxazole) belong to the class of synthetic antibiotic. Sulfonamides are widely used in veterinary medicine as feed additives and the treatment of bacteria but in humans they are usually used in combination with trimethoprim for chlamydia, respiratory and urinary tract infections (Hendricks and Pool, 2012; Pazda et al., 2019). *Sul1* is a resistant dihydropteroate synthase which mediates tolerance to a broad group of sulfonamide antibiotics (Wallmann et al., 2021). This gene is frequently found in both transposons and plasmids of Gram-negative enterobacteria but also in environmental pathogens like *Pseudomonas aeruginosa* (Pazda et al., 2019; Wallmann et al., 2021). Resistance to trimethoprim is due to the plasmid although the genes are usually found on the chromosome (Pazda et al., 2019). The *sul* genes were present in China (WTP1, WWTP2, WWTP3, WWTP4), Germany (WWTP5), Namibia (NGWRP) and United States (WRP, WRPF) as can be seen in Table 1.

Another antibiotic that can persist for relatively long periods in the absence of sunlight and is less mobile, is Tetracycline (TET; Koutsoumanis et al., 2021). TET is a naturally sourced antibiotic that is obtained from *Streptomyces* sp. (e.g., chlortetracycline, tetracycline and oxytetracycline) and they are also semi-synthetic antibiotics (e.g., demeclocycline and doxycycline; Pazda et al., 2019; Panja et al., 2021). The naturally sourced TET are used in the treatment of aquaculture and livestock, and they are also as medicine for humans (Pazda et al., 2019). For humans, TET is used to treat diseases such as malaria, rosacea, chlamydia, etc. while for livestock, it is administered as a growth promoter in concentrated animal feeding operations (Panja et al., 2021). TET is mostly found in WWTPs because it is released in human and animal faeces and urine, in its active form (Bockelmann et al., 2009). The TET genes found on bacterial chromosome, integron, transposons and plasmids (*tetA, tetB, tetC, tetD, tetE, tetG, tetH, tetM, tetL, tetO, tetQ, tetX, tetT, tetW, and tetS*) are responsible for the resistance of bacteria to TET antibiotics (Bockelmann et al., 2009; Defrancesco et al., 2017; Pazda et al., 2019). The TET genes were present in China (WWTP1, WWTP2, WWTP3, WWTP4) and United States (WRPF; Table 1).

### 2.3. The current position on ARBs and ARGs occurrences in south African WWTPs

South Africa is critically overburdened by two socioeconomic nemeses: intense water stress and HIV/AIDS. This suggests the frugal use of scarcely available water sources and the desperate adoption of alternative sources, such as greywater and treated wastewater, for domestic purposes and irrigation, which have been advocated for and practiced in some municipalities (Ateba et al., 2020). The reuse of water in S.A may be harmful to consumers, especially the immunocompromised (HIV/AIDS) and vulnerable population, because of the threat presented by AMR; so far, not fewer than four major AMR outbreaks have been recorded at national level. A 2014 WHO report identified Africa and South East Asia as the regions without established AMR surveillance systems (Tadesse et al., 2017). In response, S.A has redoubled efforts to track the incidence and prevalence of ARBs and ARGs in different environmental matrices and also identify their respective pathways and thresholds through the establishment of the South African Antimicrobial Resistance Strategy Framework. Although surveillance of WWTPs has not gained the momentum anticipated, snippets from recent investigations and reviews suggest there might be a lot more to uncover regarding occurrences of ARBs and ARGs in WWTPs (Igwaran et al., 2018; Mbanga et al., 2021; Mpondo et al., 2021; Mtetwa et al., 2021; Conco et al., 2022; Ogunlaja et al., 2022; Ramasamy et al., 2022) other worthy mentions are captured in Table 2. From the aforementioned studies, we observed a similar trend in occurrence and abundance of genes; moreover, genes coding for other virulence factors were also detected, which explains the persistence of their bearing organisms throughout the treatment processes of WWTPs. Strikingly, the investigation of Mtetwa et al. (2021) suggests the emergence of new classes of antibiotics as well as their resistance genes in WWTPs, which stem from the treatment of HIV/AIDS associated infections. This already casts a cloud of bewilderment on management of the known, and triggers anxiety regarding the unknown yet impending dangers of AMR incidences in SA.

**Table 2:** Percentage (%) resistance of bacteria to antibiotics in different WWTPs.

**Antibiotic**	**WWTP**	**% Resistance of THB to antibiotic in the influent**	**% Resistance of THB to antibiotic in the effluent**	**% Resistance of *E. coli* to antibiotic in the influent**	**% Resistance of *E. coli* to antibiotic in the effluent**	**References**
Erythromycin	WWTP14	2.5	4.8			Ateba et al., 2020
	WWTP15	5.4	3.1			Ateba et al., 2020
Ciprofloxacin (CIP)	WWTP14	2.5	1.6			Ateba et al., 2020
	WWTP15	2.6	1.6			Ateba et al., 2020
	NWWTP			17	23	Pillay and Olanirano, 2016
	NGTW			25	40	Pillay and Olanirano, 2016
Trimethoprim/sulfamethoxazole (STX)	WWTP9			70	90	Gumede et al., 2021
	WWTP10			60	100	Gumede et al., 2021
	WWTP11			40	90	Gumede et al., 2021
	WWTP12			90	90	Gumede et al., 2021
Trimethoprim	WWTP14	42.5	73			Ateba et al., 2020
	WWTP15	50	46.9			Ateba et al., 2020
Tetracycline (TET)	WWTP9			30	80	Gumede et al., 2021
	WWTP10			60	100	Gumede et al., 2021
	WWTP11			20	100	Gumede et al., 2021
	WWTP12			80	90	Gumede et al., 2021
	NWWTP			63	86	Gumede et al., 2021
	NGTW			41	68	Gumede et al., 2021
	WWTP14	10	16.1			Ateba et al., 2020
	WWTP15	40.5	17.2			Ateba et al., 2020
Penicilin	WWTP13			70	100	Ateba et al., 2020
	WWTP14	42.9	58.8			Ateba et al., 2020
	WWTP15	70.4	39.5			Ateba et al., 2020
Ampicillin	WWTP6	19	3			Turolla et al., 2018
	WWTP7	25	14			Turolla et al., 2018
	WWTP8	16	23			Turolla et al., 2018
	WWTP9			90	90	Gumede et al., 2021
	WWTP10			90	100	Gumede et al., 2021
	WWTP11			80	100	Gumede et al., 2021
	WWTP12			100	100	Gumede et al., 2021
	NWWTP			80	57	Pillay and Olanirano, 2016
	NGTW			53	60	Pillay and Olanirano, 2016
	WWTP13			0	40	Nzima et al., 2020
	WWTP14	53.6	61			Nzima et al., 2020
	WWTP15	92.6	55.8			Nzima et al., 2020

## 3. Removal of ARBs and ARGs in WWTPs

### 3.1. Removal from the influent of WWTPs

ARBs and ARGs are transported to WWTPs from ground or surface water, animal and human microbiota and mainly through sewage systems from health care facilities where antibiotics are consumed the most (Barancheshme and Munir, 2018). Health care services wastewater is the result of the residue collection from sewage of outpatients and those in the wards, kitchen and laundry, cooling and heating processes, and laboratorial discharge from the research centres and clinics (Giannakis et al., 2017). All these residues contain many substances, such as disinfectants, organic compounds, therapeutic metals, antibiotics, ARBs and ARGs which are transported to WWTPs without being preliminarily disinfected (Giannakis et al., 2017; Barancheshme and Munir, 2018). These macro-pollutants and micropollutants arriving in WWTPs have different compositions and they are of a size range (μg or ng). Which affect the solubility, volatility, adsorbability, absorbability, biodegradability, polarity, and stability of WWTPs, hence the failure of treatment by conventional WWTPs (Giannakis et al., 2017). This is seen in many developing nations, including SA, which turn WWTPs into unintentional collection points for ARBs and ARGs as they currently have no defined regulations regarding management of hospital wastes before they are disposed into the municipal WWTPs (Ben et al., 2019; Rodriguez-Molina et al., 2019). Practitioners have suggested pre-treatment while others suggest that hospital wastewater be treated onsite as a separate entity with conventional disinfectants such as chlorine, to effectively reduce bioaccumulation, and to importantly eliminate ARBs and ARGs as they can directly threaten developing countries drinking water sources (Giannakis et al., 2017; Barancheshme and Munir, 2018). However, some techniques have been employed by WWTPs in selected countries, worldwide, to detect and reduce the occurrences of ARBs and ARGs in influents (Table 3).

**Table 3:** Treatment processes of ARB’s and ARG’s in different WWTPs worldwide.

**Country (Province/ City)**	**Site of influent**	**Capacity of treatment plant**	**Year collected**	**Method of disinfection**	**Method of quantification**	**References**
China (Northern China)	WWTP1	540,000 *m^3^ / day*	November – December 2011 and June–July 2012	Anaerobic and anoxic lagoons, conventional activated sludge and Chlorination	DNA extraction and Quantitative Polymerase Chain Reaction (qPCR)	Mao et al., 2015
	WWTP2	580,000 *m^3^ / day*	November – December 2011 and June–July 2012	Anaerobic and anoxic lagoons, conventional activated sludge and Chlorination	DNA extraction and qPCR	Mao et al., 2015
China (Xi’an)	WWTP3	Not mentioned	March – June 2013	Anoxic/anaerobic/ oxic process (Ultra violet (UV) Irradiation Experiment)	DNA extraction and PCR	Zhang et al., 2017
China	WWTP4	Not mentioned	Not mentioned	Oxidation ditch process Secondary clarifier (Electrochemical (EC) and UV Experiment)	DNA extraction and qPCR	Chen et al., 2020a
Germany	WWTP5	112,000 *m^3^ / day*	September 2016, March 2017, July 2017, and October 2017	Activated sludge treatment in combination with sedimentation (Ozonation and UV irradiation Experiments)	DNA extraction and qPCR	Jager et al., 2018
Italy (Milan)	WWTP6	432,000 *m^3^ / day*	Not mentioned	Sand filtration and Peracetic acid (PAA)	Not mentioned	Turolla et al., 2018
	WWTP7	354,600 *m^3^ / day*	Not mentioned	Sand filtration and UV Radiation	Not mentioned	Turolla et al., 2018
	WWTP8	177,000 *m^3^ / day*	Not mentioned	Sand filtration and Sodium hypochloride (NaOCl)	Not mentioned	Turolla et al., 2018
Namibia (Windhoek)	New Goreangab Water Reclamation Plant (NGWRP)	21,000 *m^3^ / day*	September 2018	Pre Ozonation, Coagulation, Flotation, Dual Media Filtration, Main Ozonation, Activated Carbon, Ultrafiltration and Chlorination	DNA extraction and qPCR	Wallmann et al., 2021
South Africa (Kwazulu-Natal Durban)	Northern Wastewater Treatment Plant (NWWTP)	70,000 *m^3^ / day*	March– August 2012	Chlorination	DNA extraction and PCR	Pillay and Olanirano, 2016
South Africa (Kwazulu-Natal Durban)	New Germany Treatment Works (NGTWs)	700 *m^3^ / day*	March– August 2012	Chlorination	DNA extraction and PCR	Pillay and Olanirano, 2016
South Africa (Kwazulu-Natal Durban)	WWTP9 (Msunduzi local municipality)	76,000 *m^3^ / day*	April 2020	Aeration basins, clarifiers and chlorination.	DNA extraction and PCR	Gumede et al., 2021
	WWTP10 (uMngeni local municipality)	5,600 *m^3^ / day*	April 2020	Aeration basins, clarifiers and chlorination.	DNA extraction and PCR	Gumede et al., 2021
	WWTP11 (Surrounding suburbs)	540 *m^3^ / day*	April 2020	Aeration basins, clarifiers and chlorination.	DNA extraction and PCR	Gumede et al., 2021
	WWTP12 (Msunduzi local municipality)	500 *m^3^ / day*	April 2020	Aeration basins, clarifiers and chlorination.	DNA extraction and qCR	Gumede et al., 2021
South Africa (Kwazulu-Natal Durban)	WWTP13	Not mentioned	May–July 2017	Chlorination	DNA extraction and Multiplex PCR (M-PCR)	Nzima et al., 2020
South Africa (North West)	WWTP14	Not mentioned	2016 and 2017	Coagulation, flocculation, sedimentation, sand filtration, and chlorination	DNA extraction and PCR	Ateba et al., 2020
	WWTP15	Not mentioned	2016 and 2017	Sand filtration and chlorination	DNA extraction and PCR	Ateba et al., 2020
United States of America (Los Angeles)	Water Reclamation Plant (WRP)	235,000 *m^3^ / day*	Not mentioned	Sedimentation, nitrification, denitrification, flocculation, filtration and, chlorination	DNA extraction and qPCR	Quach-Cu et al., 2018
United States of America (Oak Creek, WI)	South Shore Water Reclamation Plant Facility (WRPF)	Not mentioned	Not mentioned	Primary clarifier (anaerobic membrane bioreactors (AnMBRs) experiment)	DNA extraction and qPCR	Singh et al., 2022

### 3.2. Removal from the effluent of WWTPs

#### 3.2.1. Chlorination

Chlorination is the most common method of choice for the disinfection of water and wastewater worldwide due to its low cost (Pillay and Olanirano, 2016; Anthony et al., 2020; Barbosa et al., 2021), its largely known technology and proven effective disinfection of a great variety of pathogenic microorganisms (Beber De Souza et al., 2015; Feng et al., 2022). But chlorination alone is inadequate for the disinfection of wastewater because it does not permanently damage ARB/ARG and it results in high regrowth of bacteria (Pillay and Olanirano, 2016; Anthony et al., 2020) as is evident with WWTP9-14, NWWTP and NGTW plant in Table 4. WWTP15 was the exception that led to a reduction of the resistance of bacteria to antibiotics. This was due to the fact that WWTP15 was actually a dam that received surface water, not waste water isolates originating from areas of high antibiotic use (Ateba et al., 2020). In the NGWRP plant, chlorination was used as a stabilization measure, preventing the regrowth of ARBs in storage tank and the drinking water distribution system (Wallmann et al., 2021). WWTP1 was also reported to have removal efficiencies of 41 ± 5%, 42 ± 3%, 69 ± 7% and 55 ± 6% for TET resistant, sulfonamide-resistant, CIP-resistant ethromycin-resistant bacteria, respectively. While in WWTP2, the removal efficiencies were 79 ± 6%, 65 ± 5%, 77 ± 8% and 55 ± 6% for TET resistant, sulfonamide-resistant, quinolone-resistant and macrolides-resistant bacteria, respectively. This is because chlorination initially lowers the total load of microbes, while significantly increasing the level of ARBs (Anthony et al., 2020; Chen et al., 2020b).

**Table 4:** Reduction of the percentage resistance of bacteria to antibiotics.

	**Disinfection**	**ERM**	**QN(CIP)**	**STX**	**TRIM**	**TET**	**PEN**	**AMP**
**WWTP6**	PAA							16%
**WWTP7**	UV							11%
**WWTP8**	NaOCl							7% INC
**NWWTP**	Chlorination		6% INC			23% INC		23%
**NGTW**	Chlorination		15% INC			27% INC		7% INC
**WWTP9**	Chlorination			20% INC		50% INC		0%
**WWTP10**	Chlorination			40% INC		40% INC		10%
**WWTP11**	Chlorination			50% INC		80% INC		20%
**WWTP12**	Chlorination			0%		10% INC		0%
**WWTP13**	Chlorination						30% INC	40% INC
**WWTP14**	Chlorination	2.3% INC	0.9%		7.5% INC	6.1% INC	15.6% INC	7.4% INC
**WWTP15**	Chlorination	2.3%	1%		27.9%	23.3%	30.9%	36.8%

(INC=Increase).

Chlorine increases cell membrane permeability by causing impairment to the cell membrane and cytoplasm (Anthony et al., 2020; Feng et al., 2022), and then directly inactivating ARGs (Gomes et al., 2019). However, when the disinfection dose is not enough, bacteria become injured and enter a viable but non-culturable state (VBNC). The injured bacteria have low metabolic activity which become active under certain conditions. They receive a large amount of DNA released from sensitive bacteria surrounding them, making horizontal transfer happen more frequently (Pillay and Olanirano, 2016; Jin et al., 2020; Feng et al., 2022). Also, when *E. coli* is exposed to chlorine it induces a specific set of proteins, making them less susceptible to disinfection (Luukkonen et al., 2014; Ateba et al., 2020). In WWTP1 the *ermB*, *sul*, *tet* and *qnr* genes were reduced by 99.8, 98, 96 and 99%, respectively. In WWTP2 the *ermB*, *sul*, *tet* and *qnr* genes were reduced by 100, 93, 85 and 96% respectively, as can be seen in Table 5. WWTP1 with a lower capacity, reduced more genes than WWTP2 with a larger capacity and hence more antibiotic residues which facilitate the maintenance and propagation of ARGs in WWTPs (Mao et al., 2015; Magureanu et al., 2021). Dosage also might have played a role in more genes being inactivated because there was an instance whereby 30 mg/l of chlorine were required for the removal of 90% of ARBs and ARG, while only 3 mg/l of ozone was required for the same reduction (Gomes et al., 2019), which is a shortcoming as chlorination forms harmful by-products, such as halo-organics (Luukkonen et al., 2014; Anthony et al., 2020) and de-chlorination is required before release to the environment as chlorine is toxic to the water life (Beber De Souza et al., 2015). In the WRP the *sul* and *bla*_SHV/TEM_ genes were both reduced by 100%. This is because of the combination of the tertiary filtration and chlorine disinfection, which produced a synergistic effect resulting in additional removal of extracellular ARGs compared to chlorine treated pre-filtered samples (Quach-Cu et al., 2018). Furthermore, the concentration of chlorine used was 25 mg/l, which is much higher than the concentration used in practice, which rarely exceeds 2 mg/l (Umar, 2022).

**Table 5:** Removal efficiency of ARGs by different disinfection methods.

	**Disinfection**	***ermB***	***sul***	***tet***	***qnr***	***bla***_***SHV/TEM***_
**WWTP1**	Chlorination	99.8%	98%	96%	99%	
**WWTP2**	Chlorination	100%	93%	85%	96%	
**WWTP3**	UV		96%	86%		
**WWTP4**	UV		99%	62%		
**WWTP4**	UV and EC		100%	97%		
**WWTP5**	UV	30.2%	30%			50% INC
**WWTP5**	Ozone	98.1%	95%			91%
**WWTP5**	UV and Ozone	98%	96%			91%
**NGWRP**	Main Ozonation, Activated Carbon and Ultrafiltration	100%	100%			
**WRP**	Filtration and Chlorination		100%			100%
**WRPF**	AnMBR	100%	100%	99%		

#### 3.2.2. Ozonation

Ozone is a bluish gas with a pungent smell and is an extremely reactive and unstable allotrope of oxygen. Ozone has been widely used in water treatment since the 19th century (Rekhate and Srivastava, 2020). Ozone is a powerful oxidant that is able to inactivate a wide range of pathogens, such as bacteria, including its spores, viruses, protozoa, and prion protein but gram-positive bacteria are less susceptible to ozone as compared to gram negative bacteria (Gomes et al., 2019; Anthony et al., 2020). This is because zone primarily diffuses to the membrane and then penetrates it, generating increased permeability (Gomes et al., 2019; Wallmann et al., 2021; Feng et al., 2022). Ozone forms reactive oxygen species (ROS) which impact the metabolism of bacteria by oxidising critical enzymes in bacterial cells, destroying their genetic material and eliminating bacterial cellular function, ultimately leading to bacterial death (Jager et al., 2018; Feng et al., 2022). The disinfection efficiency of ozone depends on the water quality, the contact time and the ozone concentration. In WWTP5, a dose of 1 g ozone per g dissolved organic carbon (DOC) led to the decrease of *ermB*, *sul* and *bla*_TEM_ genes of 98.1, 95 and 91%, respectively (Jager et al., 2018), as can be seen in Table 5. There is a clear removal effect of ARGs by ozone but less of an effect is seen on *tet* and *sul* resistant genes (Feng et al., 2022). For 100% *sul* resistant gene removal, a dose of 3–3.5 g ozone/g DOC was applied which is 3–7.5 times higher than what is usually applied at WWTPs (Wallmann et al., 2021). The doze of ozone that is required for an effective disinfection is generally higher than the one leading to organic compounds degradation and chemical micropollutants removal. The high dose demand especially when the water comprises high amounts of organic matter and solids, adds to ozone’s operational cost (Gomes et al., 2019). The combination of UV and ozone at the same time was also tested in WWTP5 but the treatment did not result in a more effective reduction compared to ozone treatment. The *ermB*, *sul* and *bla*_TEM_ genes were reduced by 98, 96 and 91%, respectively (Table 5; Jager et al., 2018). Due to bromate and the dangerous by-products during the partial oxidation of dissolved organic compounds formed after ozone treatment, UV light was not able to interpenetrate the ozone treated wastewater (Jager et al., 2018; Gomes et al., 2019). At the NGWRP plant, activated carbon and ultrafiltration were subsequently applied after ozonation. Activated carbon did not remove the ARG but instead the gene abundance returned to the value upstream of the main ozonation. Ultrafiltration then reduced the *sul* genes again to below LOD by a membrane cut-off of 40 nm but ultrafiltration tends to be non-destructive in nature, resulting in the retentate water having higher concentrations of ARBs and ARG than the influent (Wallmann et al., 2021).

#### 3.2.3. Ultraviolet radiation (UV)

UV disinfection is a well-known process used for the inactivation of pathogens (Gomes et al., 2019). UV inactivates ARGs by impairing the synthesis of RNA and DNA replication and leading to cell death, when it is absorbed by pyrimidine and purine nucleobases which cause DNA mutations (Barbosa et al., 2021). Its efficiency however depends on the type of microorganism considered as viruses and bacteria spores are the most resistant to inactivation by it, followed by intestinal protozoa, and lastly, by bacteria (Gomes et al., 2019). In WWTP7 the percentage resistance of THB resistance to AMP was reduced by 11% under a UV disinfection of 150–300 mJ cm^−2^ as can be seen in Table 4, because of THBs high tolerance to UV (Turolla et al., 2018). The concentration of the *sul* and *ermB* were reduced by only 30 and 30.2% respectively, in WWTP5 and *bla_TEM_* even increased by 50% after UV disinfection (Jager et al., 2018), as can be seen in Table 5. With only UV irradiance of 20 mJ cm^−2^ the *sul* and *tet* genes were reduced by 99 and 62%, respectively, in WWTP4 as can be seen in Table 5 (Chen et al., 2020a). While in WWTP3, the UV resulted in a reduction of the *sul* and *tet* genes by 96 and 86%, respectively (Zhang et al., 2017). More *sul* genes were reduced than *tet* genes because *tet* genes are more resistant to UV (Jager et al., 2018).

The dose of UV exposure was increased from 80 to 400 mJ cm^−2^ which completely inactivated ARBs but a high concentration of ARGs remained and the relative abundance of ARGs increased as UV dose increased (Zhang et al., 2017). This is because wastewater has high turbidity that influences the reduction efficiencies of UV, preventing the interpenetration of the UV light through wastewater. Further processing steps such as filtration for the removal of particles will enable attack of the residual contaminants by UV treatment (Jager et al., 2018). The high dose required to achieve complete removal of ARGs would be impractical in WWTPs with a high concentration of ARGs (Zhang et al., 2017). For the complete inactivation of ARBs and ARGs to happen with UV treatment, a secondary residual disinfectant is usually required (Gomes et al., 2019). This was evident with UV with a low fluence of 20 mJ cm^−2^, was able to remove all of the extracellular ARGs and reduce the *sul* and *tet* genes by 100 and 97%, respectively. When it was treated subsequently with Electrocoagulation (EC) with a current density of 20.0 mA/cm^2^ at pH 7 for 60 min.

EC is the most commonly used Electrochemical disinfection technology which is eco-friendlier and more cost-effective compared with conventional disinfection methods. When iron-based EC is applied at pH 7.0, insoluble Fe(OH)_2_ or Fe(OH)_3_ species are released, which are responsible for the removal of ARBs and ARGs. However, for EC to achieve a higher removal efficiency of ARGs, a higher current density would have to be applied, so that more hydroxides flocs can be formed (Chen et al., 2020a). PAA and NaOCl were tested as alternatives to UV but ARGs were effectively reduced by PAA rather than by NaOCl and UV radiation. Because PAA acts selectively on AMP resistant bacteria but UV and NaOCl do not act selectively on AMP resistant bacteria, displaying their disinfecting action with the same intensity on the whole bacterial community present. ARGs can also be effectively reduced by PAA rather than by UV radiation and NaOCl disinfection because PAA acts selectively on resistant micro-organisms, behaving as an effective barrier against ARBs spread into the environment (Turolla et al., 2018). UV disinfection continues to be applied wildly around the world, in spite of all its disadvantages, including its expensive equipment (Gomes et al., 2019). Table 6 Summarises the advantages and disadvantages of the treatment methods discussed.

**Table 6:** Pros and cons of treatment methods.

**Treatment method**	**Advantages**	**Disadvantages**
Chlorination	It is low in cost (Pillay and Olanirano, 2016; Anthony et al., 2020; Barbosa et al., 2021)	When *E. coli* is exposed to chlorine it induces a specific set of proteins, making them less susceptible to disinfection (Ateba et al., 2020)
	Chlorine diffuses into the intracellular component of the cell causing impairment to the cell membrane and cytoplasm (Anthony et al., 2020)	It does not permanently damage ARB/ARG and it results in high regrowth of bacteria (Pillay and Olanirano, 2016; Anthony et al., 2020)
EC	Eco-friendly and affordable (Crini and Lichtfouse, 2019; Chen et al., 2020a).	A higher current density would have to be applied for EC to achieve a higher removal efficiency of ARGs (Chen et al., 2020a).
	pH control is not necessary (Crini and Lichtfouse, 2019)	Requires post-treatment to remove high concentrations of iron ions and the sludge treatment is costly (Crini and Lichtfouse, 2019)
Membrane	It traps undissolved ARGs (Anthony et al., 2020)	There is minimal ARG removal when dissolved ARGs pass through the pores (Anthony et al., 2020)
	Anaerobic membrane bioreactors (AnMBRs) do not require energy for aeration and they produce less solids than aerobic system (Kappell et al., 2018).	Ultrafiltration tends to be non-destructive in nature, resulting in the retentate water having higher concentrations of ARBs and ARG than the influent, resulting in fouling (Ebomah and Okoh, 2020; Wallmann et al., 2021)
	AnMBRs produce methane that could be recovered for energy (Kappell et al., 2018).	Removal efficiency of membrane systems depends on the shape of the ARB, i.e., round shaped ARBs will be efficiently retained while rod shaped ARBs will be unretained on the permeate (Anthony et al., 2020)
	No chemicals required (Crini and Lichtfouse, 2019)	Investment costs are often too high for small and medium industries. With high energy requirements, maintenance and operation costs (Crini and Lichtfouse, 2019)
		Limited flow rates (Crini and Lichtfouse, 2019)
Ozone	Gram negative bacteria are more susceptible to ozone (Anthony et al., 2020).	Gram-positive bacteria are less susceptible to ozone (Anthony et al., 2020).
	It induces oxidative stress responses in surviving wastewater populations (Jager et al., 2018)	The reduction efficiency of oxidative treatments is different in different species because of the presence of anti-oxidative mechanisms in those species. The disinfection efficiency of ozone depends on the water quality, the contact time and the ozone concentration (Jager et al., 2018).
	The oxygen radicals interact with the cell surface (Ebomah and Okoh, 2020; Wallmann et al., 2021)	The oxygen radicals rarely oxidize the interior contents of the cells (Wallmann et al., 2021)
	Generation of ozone on-site (no storage-associated dangers) (Crini and Lichtfouse, 2019)	Short half-life (Crini and Lichtfouse, 2019)
UV	UV inactivates ARGs by impairing the synthesis of RNA and DNA replication and leading to cell death (Barbosa et al., 2021)	UV tends to reduce the absolute abundance and increase the relative abundance of some ARGs and it also induces bacteria into VBNC state (Jia et al., 2021)
	UV causes a selective change in the inhibition zone diameters of ARBs (Hasan Abusaiba and Al-Harmoosh, 2020; Wallmann et al., 2021)	tet genes are more resistant to UV treatment (Jager et al., 2018)
		UV light cannot interpenetrate wastewater that has high turbidity (Jager et al., 2018)

## 4. Factors affecting the efficiency of WWTPs for removal of ARBs and ARGs from wastewater

### 4.1. Biotic factors

Biological processes create an environment that is conducive for the development and spread of ARBs and ARGs (Umar, 2022). The most influential biotic factors that play an integral role in shaping compositional variations of the resistomes, in the influent and effluent of WWTPs are the morpho-functional metabolic and genetic factors of the indwelling bacterial community (Ju et al., 2019). For instance, biological removal of organic material from WWTPs is linked to the fast growth of bacteria and other microorganisms. At steady state after flocculation and the subsequent settling of biomass that is aggregated, the biomass is continuously removed as biological sludge. The sludge contains ARBs and ARGs that were present in the biomass (Uluseker et al., 2021). It has thus been suggested that part of the reduction ARGs in WWTPs is because of the removal of biomass. It was also shown that reduction of biomass positively correlated to the reduction of tetA and tetB (Du et al., 2015). Moreover, bacteria biofilm formation has been observed as a very critical mechanism to ensure the resistance to environmental pressures and stress posed by treatment methods in the WWTP. Here, secretion of certain molecules, such as adhesins and exopolymeric substances, or the extracellular appendages (pilli and fimbrae) that enable their adhesion to biosolids and sludge. A large amount of ARBs and ARGs are removed with the sludge because they stick to inorganic and organic particle in WWTPs which are released with the sludge, thus evading certain treatments or reducing contact time with certain disinfectants (Grehs et al., 2021). The activated sludge of two WWTPs in the Northern part of China have been reported to contain 30 isolates of ARGs that give resistance to macrolides, sulfonamides, tetracyclines and quinolones (Li et al., 2022; Soni et al., 2022). The dry weight of the waste sludge was also reported to contain ARGs at the rate of about 1.5 × 10^9^ to 2.2 × 10^11^ copies/g (Soni et al., 2022).

The most critical genetic factors that influences WWTP efficiency is the mobile genetic elements; this is because they influence bacterial evolution and adaptability (Li et al., 2022). However, of particular importance regarding antimicrobial resistance in WWTPs are Class 1 integrons (*intI1*), conjugal transfer protein, and resolvase (Ju et al., 2019). The central players in resistance dissemination are *intI1* which are one of the 10 ARGs markers (Ju et al., 2019; Igere et al., 2020; Ghernaout and Elboughdiri, 2020a). Different ARGs as well as the efflux pump gene qacE11 are encoded by the resistance cassettes within integrons. When one antibiotic applies selection pressure, it can select for ARGs related with multiple antibiotics within the gene cassette of the integron. This is because of the ability of the multi-gene cassettes to help co-selection and to encode different ARGs (Barancheshme and Munir, 2018). Upon conjugative plasmid transfer *intI1* get activated, allowing host bacteria to quickly develop antibiotic resistance (Barancheshme and Munir, 2018; Ju et al., 2019; Igere et al., 2020). Moreover, *intI1* has been suggested as a general indicator of resistance since plasmid-mediated resistance in microbes and metal resistance has been reported in WWTPs. This is because the WWTP environment constantly changes and bacteria are exposed to chemical stress by antibiotics, heavy metals and or both, resulting in the co-selection of ARGs increasing (Ju et al., 2019; Igere et al., 2020; Umar, 2022). Hence the increase of *intI1* being reported in the effluents of WWTPs (Umar, 2022).

### 4.2. Abiotic factors

There are numerous abiotic factors that facilitate resistance in WWTPs such as salinity, temperature, oxidation reduction potential, pH and electric conductivity (Igere et al., 2020). In a WWTP that uses activated sludge system, an imbalance of antimicrobial agents in the waste, inorganic nitrogen, pH and dissolved oxygen play an additional role in the proliferation of resistome (Ju et al., 2019; Igere et al., 2020). The oxygen and nutrients are substantially consumed by activated sludge biomass and may thus act as driving forces for both community and resistome composition (Ju et al., 2019). Antibiotics become susceptible to abiotic transformation when they are exposed to temperature, pH, and light. β-lactams are sensitized to degradation by the heat, light, and extreme pH, while methanol and water rapidly hydrolyse them. Therefore, despite their dominant presence in sewage influent, β-lactams are not detected in WWTP effluent (Bombaywala et al., 2021). Many ARBs are strictly aerobic or facultative bacteria, neutrophilic, mesophilic, and chemoorgano heterotrophic, and their ability to thrive and multiply is determined by the adequate balance of all these variables (Manaia et al., 2018). Change in season has been shown to result in fluctuation of antibiotics and ARGs in WWTPs. During spring there are higher release loads of ARGs and ARBs than those during winter months in effluent of WWTPs. The abundance of ARGs of tetracycline, sulfonamides, and vancomycin were abundantly higher in winter than in summer, while *mecA*, *tetA*, and *tetB* do not change with seasons (Du et al., 2015). High temperatures or high pH are said to be more effective for the removal of ARGs or *intI 1* than conventional treatments or low temperatures. Plausibly, the conditions that will contribute to the removal of ARBs and ARGs are temperature or pH values veering from the neutral pH range and temperatures where most ARBs thrive (Manaia et al., 2018). Most of the studies focus on the comparison of disinfection processes: hence there is no evidence gathered of a specific measurement of a bio-physico-chemical condition or factor that can result in the inactivation of ARBs and ARGs in WWTPs (Manaia et al., 2018; Pallares Vega et al., 2019).

## 5. Advanced oxidation processes (AOPs): A better alternative for removing ARBs and ARGs in WWTPs

Advanced oxidation processes (AOPs) are a set of chemical processes that are able to decompose recalcitrant organics or persistent organic compounds, pharmaceuticals and heavy metals from wastewater which conventional technologies cannot degrade (Foster, 2017; Giannakis et al., 2017; Tichonovas et al., 2017; Rekhate and Srivastava, 2020; Courti et al., 2022) and they also enhance the biodegradability of wastewater (Giannakis et al., 2017; Rekhate and Srivastava, 2020; Courti et al., 2022). AOPs are able to do this by producing oxygen radicals and large quantities of the powerful, non-selective (•OH) which act as the oxidative agents (Foster, 2017; Giannakis et al., 2017; Tichonovas et al., 2017; Rekhate and Srivastava, 2020; Courti et al., 2022). The different types of AOPs which are discussed below are Fenton based reactions, UV-based reactions, Ozone based reactions (Giannakis et al., 2017; Rekhate and Srivastava, 2020; Courti et al., 2022) and plasma-based reactions (Courti et al., 2022).

### 5.1. Theory and mechanism of Fenton

Fenton oxidation is one of the AOPs widely used for wastewater and water treatment that was discovered in 1894 and named in honour of Fenton H.J.H (Giannakis et al., 2017; Chen Y. et al., 2020; Akbari et al., 2021; Bracamontes-Ruelas et al., 2022). Fenton discovered that the reaction of ferrous (Fe^2+^) salts with hydrogen peroxide (H_2_O_2_) could oxidize tartaric acid (Bracamontes-Ruelas et al., 2022). During Fenton process, H_2_O_2_ and Fe^2+^ salts (catalyst) are added into wastewater at the same time under acidic conditions to produce reactive oxygen species such as superoxide radicals (O_2_^.-^) and singlet oxygen (^1^O_2_) and •OH (Chen Y. et al., 2020; Chen et al., 2020b; Akbari et al., 2021; Bracamontes-Ruelas et al., 2022). •OH, is the main species that plays the important part of degradation of organic pollutants and the elimination of ARBs and ARGs (Chen Y. et al., 2020; Cuerda-Correa et al., 2020; Chen et al., 2020b). When Fenton treats ARB, the cell surface gets distorted lead to the loss of cell permeability, swelling and rupture of cells and ultimately the leakage of cell components (Chen Y. et al., 2020). The direct oxidation by •OH results in the removal of extracellular ARGs (Giannakis et al., 2017).

The advantage of Fenton process is that it is easy to operate, high degradation of toxic compounds, Fe is abundant in nature and has low inherent toxicity, H_2_O_2_ is environmentally safe and easy to handle and it decomposes spontaneously to H_2_O and O_2_ (Giannakis et al., 2017; Chen Y. et al., 2020; Akbari et al., 2021). However, different parameters like pH, temperature and H_2_O_2_ and Fe^2+^ concentrations influence the degradation process. Fenton oxidizing method is limited to acidic condition (Akbari et al., 2021). The process is limited by the strict acidic operational condition (pH = 3–3.5) because less amount of the •OH is produced due to the formation of Fe^2+^ complexes with water at a lower pH (<2.5) or the precipitation of ferric oxyhydroxides at a higher pH (>4; Chen Y. et al., 2020; Chen et al., 2020b). Maximum reduction of 2.58 to 3.79 logs are achieved for ARGs at pH 3 as compared to 2.26 to 3.35 logs reduction at pH 7 (Michael-Kordatou et al., 2018). Depending on the nature of the pollutant and the wastewater in which it is found, this pH range may not degrade some pollutant (Bracamontes-Ruelas et al., 2022). A basic pH is not an option for Fenton oxidation because the iron would catalytically decompose H_2_O_2_ into oxygen and water, without forming •OH (Cuerda-Correa et al., 2020). The concentration of H_2_O_2_ and Fe^2+^ are the main factors that determine the inactivation efficiency of the Fenton process (Chen Y. et al., 2020; Chen et al., 2020b). Fe^2+^ is regenerated, so that the Fenton process can be regarded as catalytic with respect to iron. As the iron concentration increases, the oxidation rate of organic compounds increases as the iron concentration increases to a point at which an additional increase in iron concentration is ineffective (Cuerda-Correa et al., 2020). A study showed that a molar ratio Fe^2+^/H_2_O_2_ from 0.033 to 0.1, results in an increase in the removal efficiency. The t*etG* genes are less susceptible to Fenton oxidation as compared to *int1*. *E. coli* resistant to ampicillin, ciprofloxacin and tetracycline is completely inactivated at a ratio concentration of Fe^2+^/H_2_O_2_ 5:10 (Michael-Kordatou et al., 2018). Small increments of H_2_O_2_ dose result in evident decreases in the toxicity of the effluent once a minimum threshold has been reached (Cuerda-Correa et al., 2020). A lack of H_2_O_2_ may decrease the performance of the Fenton oxidation to treat wastewater while high concentrations of H_2_O_2_ leads to the scavenging of ·OH (Cuerda-Correa et al., 2020; Bracamontes-Ruelas et al., 2022). The reaction rate in the Fenton process increases between 20 and 40°C, the effect being more noticeable at temperatures below 20°C, the effectiveness of the reagent decreases between 40 and50°C. This is due to the accelerated decomposition of H_2_O_2_ into oxygen and water (Cuerda-Correa et al., 2020).

The disadvantage of using Fe^2+^ salts is that it results in a large amount of iron-containing sludge, which is hard to recover or remove, causing high operational cost and secondary pollution (Chen Y. et al., 2020; Cuerda-Correa et al., 2020; Akbari et al., 2021). For this reason, the homogeneous Fenton-like method was developed to reduce iron species dissolved in the environment, without critically affecting the efficiency of the process and to overcome the problems of Fenton oxidation (Cuerda-Correa et al., 2020; Akbari et al., 2021). The homogeneous Fenton-like method uses Fe^3+^ instead of the more expensive Fe^2+^ salts (Cuerda-Correa et al., 2020). The Fe^3+^ salt reacts with H_2_O_2_ which decomposes into •OH, and the Fe^3+^ is reduced to Fe^2+^, and its catalytic process occurs throughout the liquid phase (Cuerda-Correa et al., 2020; Bracamontes-Ruelas et al., 2022). For most applications, the catalyst cycle starts quickly if organic material and H_2_0_2_ are in sufficient concentration regardless of whether Fe^2+^ or Fe^3+^ is used; (Cuerda-Correa et al., 2020). A homogeneous Fenton-like process is easy to operate and is effective in terms of pollutant removal as the circular use of the catalyst reduces Iron rich sludge. However, excessive sludge is still produced and the operational pH is limited to below 3 (Cuerda-Correa et al., 2020; Akbari et al., 2021). The other disadvantage of the homogeneous Fenton-like method is that Fe^3+^/H_2_O_2_ results in slow decomposition rate of H_2_O_2_ and slower oxidation rate of organic solutes are markedly slower than when Fe^2+^/H_2_O_2_ is used (Cuerda-Correa et al., 2020).

The heterogeneous Fenton-like was developed to solve the problems presented by the homogeneous Fenton-like process (Cuerda-Correa et al., 2020; Akbari et al., 2021; Bracamontes-Ruelas et al., 2022). In heterogeneous Fenton oxidation, a reaction takes place between H_2_O_2_ and Fe (III) in different forms, e.g., Fe_2_O_3_ or α-FeOOH or zero-valent iron (ZVI), etc. (Cuerda-Correa et al., 2020; Akbari et al., 2021). If solid catalysts are used, sludge generation is reduced as physical adsorption occurs at the surface of the solid catalyst (Cuerda-Correa et al., 2020; Bracamontes-Ruelas et al., 2022). Heterogeneous Fenton-like processes is gaining importance, but it appears to be less effective than a homogeneous Fenton process due to mass-transfer limitation. (Cuerda-Correa et al., 2020). For Fenton-like processes, a high concentration of catalyst will consume •OH, limiting practical application and prevent the degradation of pollutant and therefore raising treatment costs. Low concentration of H_2_O_2_ leads to low availability or lack of •OH and reduce degradation efficiency. Excessive concentration of H_2_O_2_ is also not appropriate for removal of contaminants in Fenton-like process (Akbari et al., 2021). It should be clarified that if the wastewater contains heterogenous cocktails apart from the target pollutant to be treated, the other contaminants may decrease the removal performance of the target pollutant since Fenton AOP is a non-selective treatment process (Bracamontes-Ruelas et al., 2022).

### 5.2. Theory and mechanism of UV assisted

#### 5.2.1. UV/hydrogen peroxide

To effectively control HGT and ARGs, combination of UV technology with different radical promoters have been investigated. Some of the most researched AOPs include UV/chlorine, H_2_O_2_/Fe^2+^/UV (photo-Fenton), UV/H_2_O_2_, UV/O_3_, UV/peroxydisulfate (PDS), UV/peroxymonosulfate (PMS), and UV/TiO_2_ (Sharma et al., 2019; Umar et al., 2019). Among all these combinations, the most widely researched in water and wastewater treatment at small scale is UV/H_2_O_2_ (Umar et al., 2019; Umar, 2022). A few full-scale investigations have been done in recent years, using UV/H_2_O_2_ to damage ARGs (Umar, 2022). UV/H_2_O_2_ inactivates ARBs and ARGs by production of radicals by UV/H_2_O_2_ when it receives radiation energy (Grehs et al., 2021; Li et al., 2022). During photolysis two HO• radicals are produced per photon absorbed by H_2_O_2_ (Giannakis et al., 2017; Umar, 2022). The efficiency of the process strongly depends on the oxidative ability and production velocity of the •OH (Giannakis et al., 2017; Umar et al., 2019). The •OH can attack electron-rich organic contaminants at high rate constant and ultimately leads to their transformation to CO_2_ and H_2_O (Sharma et al., 2019; Umar et al., 2019). The •OH inactivates the ARBs by making the oxidation potential of the chemical system better, leading to modifications in the bacterial cell structure (Ghernaout and Elboughdiri, 2020b).

The other main factor which will affect the inactivation of ARBs and ARGs in the UV/H_2_O_2_ processes are the UV absorption, the more ARBs absorbs UV, the more internal components get damaged and hence gets inactivated (Giannakis et al., 2017; Michael-Kordatou et al., 2018). Hence, when the UV absorbance of the target pollutant is high or when strong photon absorbers are present, the efficiency of UV/H_2_O_2_ process significantly decreases (Giannakis et al., 2017). The UV fluence required for a real-water matrix with organics could be very high, therefore wastewater would have to be pre-treated prior to UV AOPs for the removal of contaminants to improve the process performance (Umar, 2022). A UV fluence of 50–130 mJ/cm2 for UV/H_2_O_2_ achieves 4 logs reduction of ARGs (Sharma et al., 2019; Ghernaout and Elboughdiri, 2020a). The UV fluence delivered to clear water is 1.4 folds higher than that delivered to wastewater (Umar, 2022). Other important factors include the concentration of H_2_O_2_ and of the target compound, the pH of the matrix, the presence/absence of scavenging compounds (e.g., bicarbonates) and the reaction time (Giannakis et al., 2017; Umar, 2022). With a concentration of 20–25 mg/l of H_2_O_2_, antibiotic resistant *E. coli* and *tetW* were deactivated after 240 min while the blaTEM gene was still present at 540 min. A pH of 3 which is practically not feasible and a high H_2_O_2_ concentration of 340 mg/l are considered best for damaging ARGs. This was evident as 2.8–3.5 logs of sul1, tetG and tetX were reduced at these conditions within 30 min, with a higher reduction of the tetracycline than sulphonamide genes as compared to 1.55 to 2.32 logs reduction at wastewater pH of 7 (Michael-Kordatou et al., 2018; Umar, 2022). The reduction of *ampC* and *mecA* was approximately 2.3–2.9- and 1.4–2.7-logs, respectively, with different concentrations of H_2_O_2_ of (340, 1700, and 3,400 mg/l) for a UV fluence of 120 mJ/cm2 (Umar, 2022). The inactivation of ARGs is generally lower than that of *E. coli* (Michael-Kordatou et al., 2018; Sharma et al., 2019).

With UV/H_2_O_2_ treatment inactivation of ARBs is faster than the damages to ARGs. An increase in pH does not have any influence on damage to ARGs by UV/H_2_O_2_ (Sharma et al., 2019). Lower amounts of i-ARGs are generally inactivated as compared to e-ARGs because of the preservative functions of cellular components versus UV and the scavenging of •OH and oxidising species by cellular components (Sharma et al., 2019; Ghernaout and Elboughdiri, 2020a). The high reaction between •OH and H_2_O leads to scavenging of •OH and hence •OH cannot penetrate the cell (Sharma et al., 2019; Umar et al., 2019). Removal of residual H_2_O_2_ after treatment could be beneficial to reduce scavenging of •OH since only approximately 5–10% of the H_2_O_2_ is used during the treatment process (Umar et al., 2019). Organic matter in complex water matrices also scavenge radicals leading to reduced oxidation efficiency of •OH and hence fairly similar rates of damage of e-ARG damage by UV-only and UV/H_2_O_2_ treatments (Umar, 2022).

### 5.3. Theory and mechanism of ozone assisted

#### 5.3.1. Ozonation and UV radiation

The different types of ozone based AOPs that are used for water and wastewater treatment are O_3_/H_2_O_2_, O_3_/UV and O_3_/UV/H_2_O_2_ (Chen Y. et al., 2020; Cuerda-Correa et al., 2020; Rekhate and Srivastava, 2020). A recent study which is apparently the first to compare the effectiveness mentioned ozone AOPs for the inactivation of ARBs from real sewage treatment plant (STP) wastewater. The study revealed that O_3_/UV resulted in better inactivation rates of ARBs than the other ozone AOPs (Chen Y. et al., 2020). There are however relatively few studies done in the literature devoted to the removal of pollutants by O_3_/UV processes in comparison with other ozone AOPs (Igere et al., 2020). In O_3_/UV the wavelength is less than 300 nm because ozone strongly absorbs UV light of wavelength λ = 254 nm (Cuerda-Correa et al., 2020; Bracamontes-Ruelas et al., 2022). The •OH are produced by the reaction of O• with H_2_O after the photolysis of ozone (Rekhate and Srivastava, 2020). The •OH can also be generated indirectly by the O_3_/UV combination. The ozone is dissolved and splits, a fast reaction of atomic oxygen (O) with H_2_O takes place, producing thermally excited H_2_O_2_ which then results in the production of •OH (Cuerda-Correa et al., 2020; Bracamontes-Ruelas et al., 2022). The •OH react with the organic substances while UV radiation speeds up the kinetics of the process. UV radiation also degrades some compounds by direct photolysis and it excites the organic molecules of the pollutant, increasing their vulnerability towards an attack by the •OH (Cuerda-Correa et al., 2020).

Within a minute, over 99% inactivation of carbapenem-resistant Enterobacteriaceae (CRE), extended spectrum β-lactamase producing Enterobacteriaceae (ESBL-E), multidrug-resistant Acinetobacter (MDRA), multidrug-resistant Pseudomonas aeruginosa (MDRP), methicillin-resistant *Staphylococcus aureus* (MRSA), and vancomycin-resistant Enterococcus (VRE) was inactivated by the O_3_/UV combination. The inactivation rates after half a minute of treatment were 94% and for CRE, 87% for ESBL-E, 32% for MDRA, 94% for MDRP, 50% for MRSA, and 94% for VRE, respectively (Chen Y. et al., 2020). The efficiency of production of •OH is however low by the O_3_/UV combination due to low photolysis efficiency (Umar et al., 2019; Bracamontes-Ruelas et al., 2022). The combination is also expensive since ozone generators and UV lamps consume large amounts of electric energy (Cuerda-Correa et al., 2020; Bracamontes-Ruelas et al., 2022). Ozone also low solubility in water which results in mass transfer limitations (Umar et al., 2019). Mostly a toxicity monitoring is required since the ozone AOPs result in problematic oxidation by-products (Ghernaout and Elboughdiri, 2020b). However, the good thing about O_3_/UV is the fact that the generation of bromate is inhibited (Cuerda-Correa et al., 2020). Depending on the pollutants to be treated in the wastewater by O_3_/UV combination, the optimum ozone dosage and radiation exposure need to be optimised, simultaneousness with pH (Bracamontes-Ruelas et al., 2022).

### 5.4. Theory and mechanism of cold atmospheric plasma

Plasma is the fourth basic state of matter after solid, liquid and gas (Ghernaout, 2020; Sanito et al., 2022). Plasma is a partially or completely ionized gas and an electroneutral mixture carrying free reactive radicals (including reactive nitrogen and oxygen species), charged particles, electrons, ions, UV photons and quanta of electromagnetic radiation (Ghernaout, 2020; Giacometti et al., 2021; Courti et al., 2022). The ionized gas is generated by applying thermal energy, or electromagnetic radiation energy, or mostly an electric field to a carrier gas at atmospheric pressure and room temperature (Von Wintersdorff et al., 2016; Giacometti et al., 2021). The reactive species may be generated in bubbles in the liquid or directly in liquid, water surfaces, aerosols and clusters (Jamroz et al., 2014; Sanito et al., 2022). Plasmas can be divided into thermal and non-thermal depending on the thermal equilibrium between the electrons (Te) and gas (Tg). Non-thermal plasma is out of thermodynamic equilibrium (Te > > Tg) while thermal plasmas are in thermodynamic equilibrium (Te = Tg; Courti et al., 2022). Non-thermal plasma is also known as cold atmospheric plasmas (CAPs), atmospheric pressure plasmas (APPs) or Non-equilibrium atmospheric pressure discharge (NEPD; Ghernaout and Elboughdiri, 2020b).

CAP is an emerging technology, that has been frequently applied in sterilization, waste water treatment., cleaning and bio-decontamination, to inactivate bacteria in water and food produce, as a fertilizer and in chemical reduction (Jamroz et al., 2014; Li et al., 2015; Royintrarat et al., 2019). CAP is one of the advanced oxidation processes (AOPs), possessing both chemical and physical processing traits (Li et al., 2015). It is however environmentally friendly as it does not inject poisonous chemicals, does not form waste or toxic by-products and it neutralizes pathogens. It also has simpler equipment which is secure and easy to operate and has higher energy efficiency (Jamroz et al., 2014; Ghernaout, 2020). CAPS can reduce the number of bacteria in both gram-negative and gram-positive bacteria as it makes use of permeabilization of the cell membrane or cell wall as one of the mechanisms of inactivation of ARBs and ARGs (Li et al., 2015; Niedzwiedz et al., 2019; Royintrarat et al., 2019). Permeabilization by overcoming the tensile strength of the membrane causing it to rupture at a point of small local curvature and ultimately leading to leakage of intracellular components. Then irreversible oxidative damage to intracellular proteins and DNA occurs, which leads to cell death, preventing the growth of ARBs and ARGs (Ercan et al., 2016; Niedzwiedz et al., 2019; Rezaei et al., 2019). This is all due to the reactive species of CAP which are classified as primary species, secondary species and tertiary species (Sanito et al., 2022).

The primary species, ROSs and RNSs oxidize nucleic acids, proteins, and lipids (Ghernaout, 2020), but ROS is the main component which reacts with lipid cell membranes and then lipid peroxides will damage DNA and protein permanently. More ROS are generated under water, which results in bacteria being killed more efficiently than those above water (Royintrarat et al., 2019). The shock waves help to mix the liquid, enhancing the efficiency of CAP in the sterilization process (Niedzwiedz et al., 2019; Rezaei et al., 2019). UV which is also a primary species that deteriorates nucleic acids (Ghernaout, 2020) and is involved in the generation of secondary species such as •OH (Niedzwiedz et al., 2019; Rezaei et al., 2019; Royintrarat et al., 2019; Anthony et al., 2020). •OH, are highly reactive oxidant agents that play the most important role in the removal of organic pollutants, oxidization of organic and inorganic compounds and as disinfectants (Beber De Souza et al., 2015; Magureanu et al., 2021).

The primary species have short lifetimes, but they contribute to degradation more than long lived ones which have a less dominant effect (Magureanu et al., 2021). When ROS and RNS dissolve in liquid, they form ozone (O_3_), hydrogen peroxide (H_2_O_2_), nitrate (NO_3_^−^) and nitric oxide (NO^−^), which are tertiary, long-lived species (Sanito et al., 2022). These species result in a decrease in the pH of the target liquid of up to 2 pH values (Von Wintersdorff et al., 2016). At low pH, species reduce the resistance of bacteria to an acidic environment, which helps in better penetration of species into the bacteria cell wall (Royintrarat et al., 2019). The long-term, post-plasma effect is mainly caused by the reaction between H_2_0_2_ and ozone during the peroxone process, that forms •OH (Magureanu et al., 2021).

When plasma is applied above liquids, a 5log reduction of bacteria and 0.19-log degradation for i-mecA genes is achieved with a low plasma influence of 0.12 kJ/cm2. To achieve 1 log reduction of i-mecA, a plasma treatment of more than 0.53 kJ/cm2 is required, while only 0.12 kJ/cm2 only could result in more than 1-log degradation of e-mecA. A plasma intensity of 0.35 kJ/cm2, reduces e- and i-mecA genes by 2.6 and 0.8 logs, respectively (Liao et al., 2018a; Courti et al., 2022). ARBs are less difficult to inactivate than ARGs. Vancomycin-resistant enterococci (VRE) count reduced by more than 5 log, below the detection limit, while vanA resistance genes remained in the order of 10^5^ copies/mL even though it showed a reduction of more than 4 log (Furukawa et al., 2022). Higher inactivation requires a longer treatment time which is proportional to the applied energy of CAP (Liao et al., 2017b). Longer time of 30 min increases gene reduction in tetA, tetR, aphA, and tnpA was increased to 5.8, 5.4, 5.3, and 5.5 log, respectively (Courti et al., 2022). To shorten the time, a higher frequency could be used. The initial voltage is the most important for inactivation of ARBs and ARGs (Furukawa et al., 2022). A higher plasmas voltage of 18 kV reduces resistant *E. coli* by approximately 6.3 log, while a lower voltage of 10 kV reduced the bacteria by 4.4 log. A higher flow rate of 2.5 l min-1 reduced resistant *E. coli* by 7 log while a flow rate of 1.5 l min-1 reduced the bacteria by only 5.5 log in 10 min (Courti et al., 2022). The fixed gap distance and the input power also affect inactivation. For example, it takes only takes 30s to inactivate S. aureus under a 2 mm gap distance at 60 W. A larger gap results in less inactivation, for instance inactivation of *E. coli* increased from 80% to 99.93% after 3 min treatment when the gap was reduced from 5 to 3 cm (Liao et al., 2017b).

#### 5.4.1. State-of-the-art of CAP

**Figure 2:**
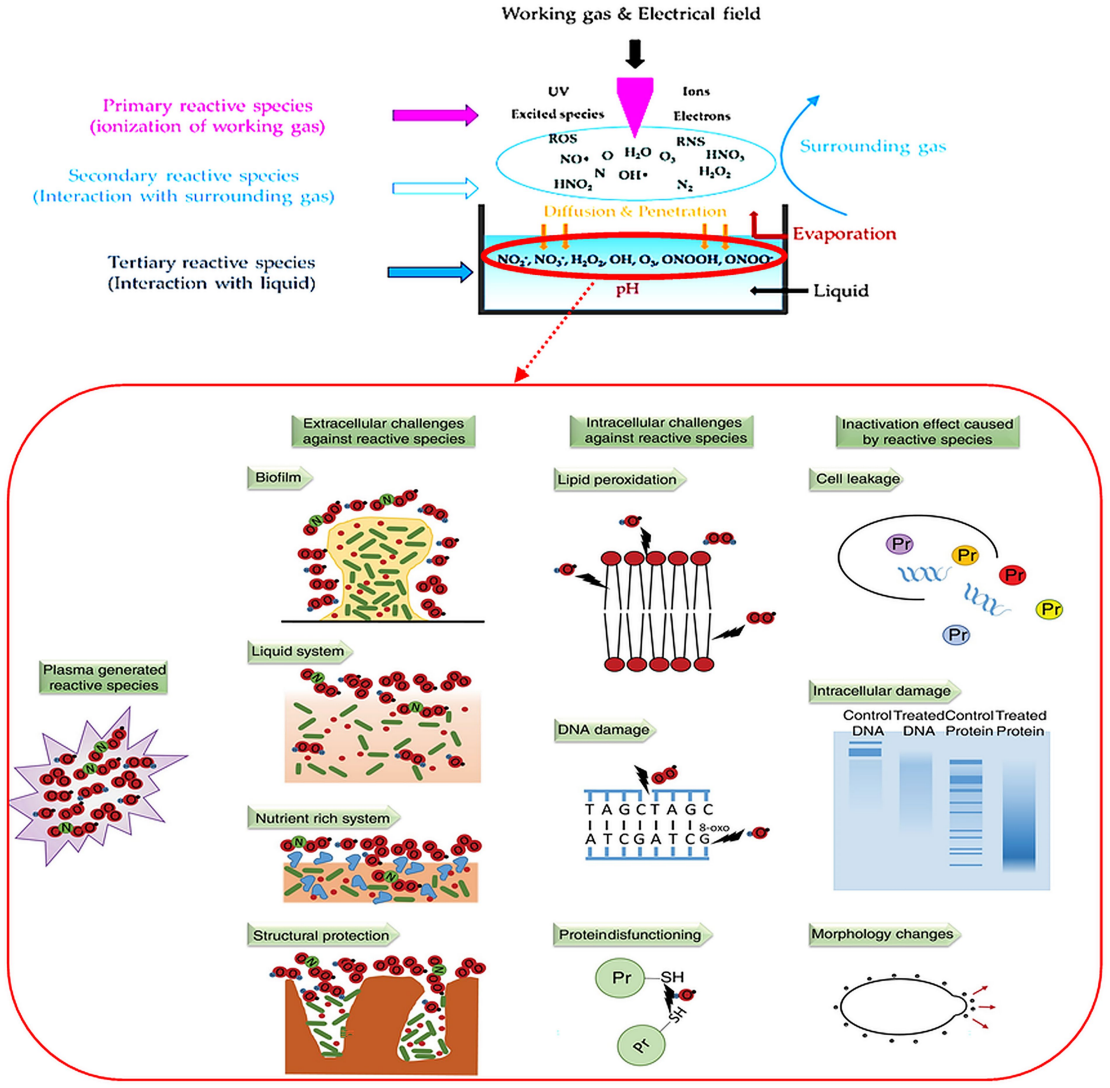
Mechanisms of plasma inactivation of ARBs and ARGs in liquid showing the reactive species released into pollutant containing aquatic matrix and their subsequent interaction with microbial communities and morphological and genetic components (Bourke et al., 2017; Courti et al., 2022).

**Figure 3:**
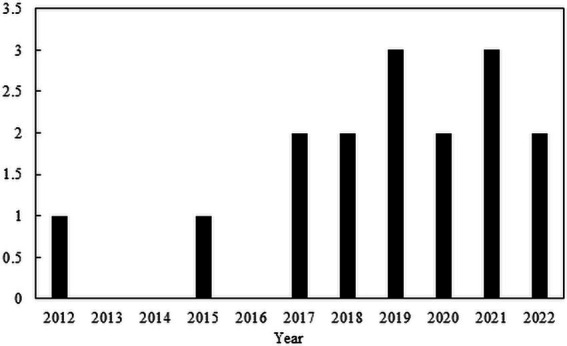
Trends articles of CAP for inactivation of ARBs and ARGs in water treatment.

The trend of the studies with the use of CAP for the inactivation of ARBs and ARGs can be seen in Figure 2, and the publications are shown in Table 7. There is not much information about CAP for the inactivation of ARBs and ARGs in wastewater. Most publications out there focused on the removal of non-resistant bacteria and wound treatments in medicine (Figure 3).

**Table 7:** Sample of studies done using CAP for treatment of wastewater.

Journal articles and patents	Year	Reference
Nonthermal Atmospheric Plasma Rapidly Disinfects Multidrug-Resistant Microbes by Inducing Cell Surface Damage	2012	Kvam et al., 2012
A comparative study for the inactivation of multidrug resistance bacteria using dielectric barrier discharge and nano-second pulsed plasma	2015	Hoon Park et al., 2015
Effect of preliminary stresses on the resistance of *Escherichia coli* and *Staphylococcus aureus* toward non-thermal plasma (NTP) challenge	2017	Liao et al., 2017a
Lethal and Sublethal Effect of a Dielectric Barrier Discharge Atmospheric Cold Plasma on *Staphylococcus aureus*	2017	Liao et al., 2017b
Application of a Dielectric Barrier Discharge Atmospheric Cold Plasma (Dbd-Acp) for *Escherichia coli* Inactivation in Apple Juice: Inactivation of *E. coli* by cold plasma…	2018	Liao et al., 2018b
Combating *Staphylococcus aureus* and its methicillin resistance gene (mecA) with cold plasma	2018	Liao et al., 2018a
Degradation kinetics of cold plasma-treated antibiotics and their antimicrobial activity	2019	Sarangapani et al., 2019
Study on the killing effect of cold atmospheric pressure plasma on MRSA *Staphylococcus aureus* *in vitro* and *in vivo* infection model	2019	Namini et al., 2019
Degradation of antibiotic resistance contaminants in wastewater by atmospheric cold plasma (ACP): Kinetics and mechanisms	2019	Liao et al., 2019
Antibiotic-Resistant and Non-Resistant Bacteria Display Similar Susceptibility to Dielectric Barrier Discharge Plasma	2020	Sakudo and Misawa, 2020
Inactivation of antibiotic resistant *Escherichia coli* and degradation of its resistance genes by glow discharge plasma in an aqueous solution	2020	Yang et al., 2020
Cumulative damage by nonthermal plasma (NTP) exceeds the defense barrier of multiple antibiotic-resistant *Staphylococcus aureus*: a key to achieve complete inactivation	2021	Liao et al., 2021
Cold Atmospheric-Pressure Plasma Caused Protein Damage in Methicillin-Resistant *Staphylococcus aureus* Cells in Biofilms	2021	Guo et al., 2021
Plasma induced efficient removal of antibiotic-resistant *Escherichia coli* and antibiotic resistance genes, and inhibition of gene transfer by conjugation	2021	Li et al., 2021
Inactivation of antibiotic resistant bacteria and their resistance genes in sewage by applying pulsed electric fields	2022	Furukawa et al., 2022
Degradation of Bacterial Antibiotic Resistance Genes during Exposure to Non-Thermal Atmospheric Pressure Plasma	2022	Courti et al., 2022

#### 5.4.2. Challenges

Although the aforementioned wastewater treatment methods in our study have demonstrated several potentials of repressing the concentrations of antibiotics, ARBs and ARGs in wastewater streams, researchers are likewise concerned about their environmental sustainability and unregulated ecotoxicology. For instance, during chlorination, disinfection intermediates or transformation products may be formed from the earliest interactions with organic and inorganic matter before subsequent contacts with the pathogenic microflora, Consequently, this phenomenon elevates the ecotoxicity of the final receiving natural water bodies (Yang et al., 2019; Li et al., 2023); worse still, it enhances the selection of chlorine tolerant bacteria as well as confer multiple resistance through the exchange of other virulence genes. As earlier mentioned, the lesser contact time with original chlorine concentrations and more contact time with by-products afford the indwelling bacteria resistance mechanisms, whereby their cell walls are at worst made porous to receive the floating DNA of other dead chlorine-susceptible ARBs species (Luo et al., 2021). Similar to chlorination, the efficiency of other treatment methods is heavily reliant on dosage, exposure time and even the physicochemical properties of the wastewater matrix. In this regard, the interactions of radicals generated from treatment techniques like AOPs, ozonation and UV radiation with the organic and inorganic matter interfere with their optimal contact with ARBs and ARGs, thereby preventing their complete inactivation. In order to achiever near complete inactivation using these methods, a high dosage and exposure would be warranted, which is not only impracticable in ARBs and ARG-laden wastewater matrices but also might distort the biogeochemical cycle of natural water bodies downstream of the WWTP, thereby creating ecological imbalance, and ultimately having adverse impacts on humans through the food-water nexus. In order to sustainably mitigate these occurrences, more environmentally and cost-friendly alternatives should be adopted, such as enzymatic biodegradation of pollutants as well as the bio electrochemical treatment of the ARB-ARGs cocktails, which could also be coupled with inexpensive acute DNA-binding filtration techniques. Moreover, optimizing the synergistic effect of extant advanced treatment technologies and green technologies would ensure well-balanced inactivation or elimination of ARBs and ARGs.

There are few publications on the study of CAP as a disinfection method for ARBs and ARGs in wastewater. The studies that have been conducted on CAP are laboratory scale (Crini and Lichtfouse, 2019), There is lack of research devoted to the upscaling to industrial level (Tichonovas et al., 2017), the efficiency may not be the same when tested in full scale WWTPs. CAP affects the properties of the exposed surfaces on the upper layer of liquids, it is impossible to store and unlike UV radiation, it has a non-remote action (Scholtz et al., 2021). Intracellular (i-)antibiotic resistance genes (ARGs) require higher plasma intensity was for degradation as compared to extracellular (e-)ARGs because of the shielding effects of the outer envelopes or intracellular components against plasma (Liao et al., 2018a, 2019).

## 6. Conclusion and future perspectives

It is evident that the urgency required for tackling antibiotic resistance cannot be overemphasized, especially due the consistent change in dynamics involved; however, the constancy of occurrence of antibiotics, ARBs and ARGs in WWTPs clearly imply the poor regulation or abuse of certain classes of antibiotics. It is therefore necessitous that drastic regulatory measures are imposed on manufacturers and healthcare settings (regarding wastewater treatment and discharge) as well as proper education of potential consumers to practice safe and ethical consumption of antibiotics.

Using the WWTP-antibiotics consumption-ARBs-ARGs nexus, it is apparent that WWTPs not only serve as sinks but also as intelligent epidemiological and community diagnostic tools; therefore, regional and global databases should be setup based on consistent research-based information, in order to assist policy makers, engineers and citizens in the campaign against the prevalence of AMR and associated medical inconveniences they may cause. Ultimately, prevention of such incidences is insufficient as there already exist copious amounts of constantly evolving ARGs and ARBs; hence the prompt intervention of wastewater treatment technologies is necessitated.

From the extant treatment techniques appraised, we observed the potential contribution of chlorination to the abundance of ARBs and ARGs, whilst reducing the total load of microbes, as well as further inducing selective pressure through the formation of harmful intermediates, such as halo-organics that correspondingly adversely impact aquatic fauna. Also, ozone, though needing a lower dose to achieve the same efficiency as chlorination is rendered ineffective by Gram-positive bacteria and the environmentally critical *tet* and *sul* genes, and hence need a higher dose to guarantee 100% removal. However, this comes at the expense of bromate production and other toxic intermediates during the partial oxidation of dissolved organic compounds. All other techniques, such as Fenton- and UV-mediated treatments have major drawbacks as their aforementioned confrere, such as secondary pollution, formation of toxic intermediates or impracticability of total removal of ARBs and ARGs.

Cold atmospheric plasma is gradually gaining espousal in water and wastewater management due to its desirable antimicrobial properties and also its ability to degrade certain microcontaminants, which antibiotics is not an exception. However, research is currently being refined at laboratory scale only; there has not been a scale up or large-scale deployment of such technologies to assist with the overwhelmed conventional WWTPs. This therefore warrants further research work to build on already existing data, thereby optimizing laboratory studies and advancing them into scale up studies.

The foundation of the future with regard to ARBs and ARGs diagnostics and remediation has already been laid for improvements thereupon. The world has gone abuzz with advancements in artificial intelligence-themed technologies, which facilitate the dissemination of molecular information of this phenomenon, through the omics (genomics, transcriptomics, metabolomics, proteomics, plasmidomics etc.) and bioinformatics, which uncover the complete information of unculturable organisms. Also, bio-nanosensors (nanotechnology) have already been developed for the early, rapid detection of minute quantities of micropollutants, which will trigger drastic actions to prevent their accumulation. Moreover, with consciousness toward a sustainable environment, nano-based biotechnology and artificial intelligence have also been attempted in the generation of electrical energy from pharmaceutical residues through the performances of bioelectrochemical systems, such as microbial fuel cells (MFCs), microbial electrolytic cells (MECs) *inter alia*. Interestingly, the manipulability of the aforementioned cells could permit the seamless integration of plasma technology, which is assumed to not only ensure the improved management of pollutants (both chemical and microbial), but also enhance its electricity generating capacity. The future anticipations of behaviour of ARBs, ARGs cannot be accurately decipherable at the moment, as these organisms and molecules constantly evolve, much to the befuddlement of research scientists, engineers and policy makers. Therefore, a robust surveillance and management scheme, involving all stakeholders should be implemented and reviewed frequently in order to increase our readiness for unsuspected episodes of AMR outbreaks.

## Author contributions

TM was the main author and responsible for the first draft of the manuscript. All authors provided review and comment on the subsequent versions of the manuscript. JU wrote some sections in the subsequent versions of the manuscript. MD, CT, and SI were the main academic and research supervisors, and also provided input, review and editing towards the manuscript. All authors contributed to the article and approved the submitted version.

## Funding

This work was supported by the Government of the United Kingdom under the Global Challenge Research Funds through the Royal Society in which the corresponding author SI is a FLAIR Fellow [FLR\R1\201683]. JU was supported by the National Research Foundation of South Africa [Grant no: 138445].

## Conflict of interest

The authors declare that the research was conducted in the absence of any commercial or financial relationships that could be construed as a potential conflict of interest.

## Publisher’s note

All claims expressed in this article are solely those of the authors and do not necessarily represent those of their affiliated organizations, or those of the publisher, the editors and the reviewers. Any product that may be evaluated in this article, or claim that may be made by its manufacturer, is not guaranteed or endorsed by the publisher.
