# Supplementary material for: Inactivation of antibiotic-resistant bacteria and antibiotic-resistance genes in wastewater streams: Current challenges and future perspectives
Source: Front Microbiol. 2023 Jan 16;13:1100102. doi: 10.3389/fmicb.2022.1100102 (PMC9888414; doi:10.3389/fmicb.2022.1100102)
Supplement: Supplementary file 1 [file Data_Sheet_1.pdf]

Supplementary Material

Inactivation of antibiotic-resistant bacteria and antibiotic-resist[illegible]e genes in wastewater streams: Current challenges and future perspectives

Thabang B. M. Mosaka[1], John O. Unuofin[1], Michael O. Daramola[1], Chedly Tizaoui[2] Samuel A. Iwarere[1]*

[1]Department of Chemical Engineering, Faculty of Engineering, Built Environment and Information Technology, University of Pretoria, Hatfield, Pretoria 0002, South Africa
[2]Water and Resources Recovery Research Lab, Department of Chemical Engineering, Faculty of Science and Engineering, Swansea University, Swansea SA1 8EN, United Kingdom

Corresponding author: samuel.iwarere@up.ac.za

Figure S1 is the flow diagram following the Preferred Reporting Items for Systematic Reviews and Meta-Analyses (PRISMA) approach used for this systematic review and compilation of removal efficiencies of antibiotic - resistant bacteria and antibiotic-resistance genes reported in actual wastewater treatment plants.

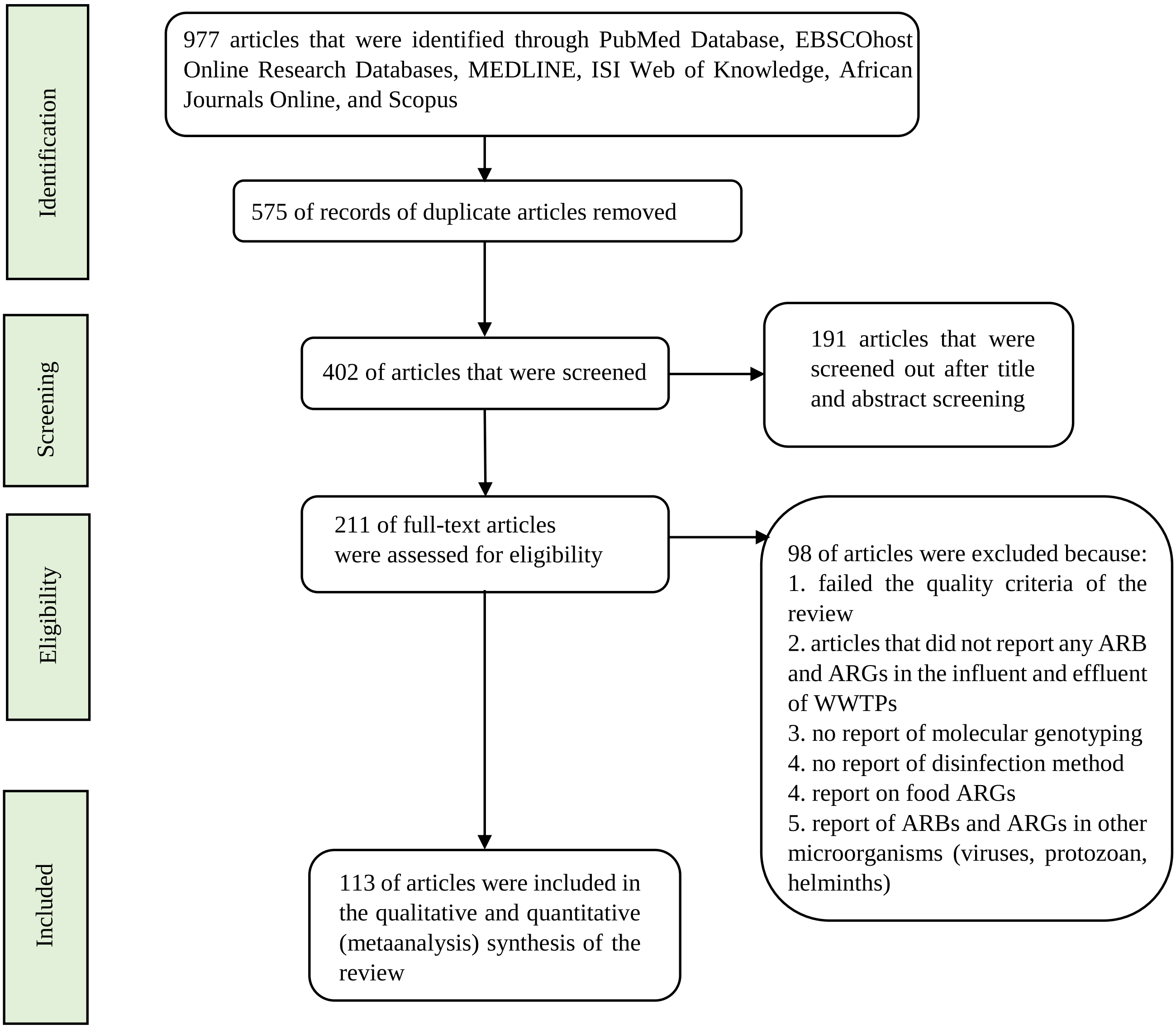

Figure 1: Flow diagram summarizing the process of literature search and selection.
